# Wearable PEDOT:PSS/DVS‐Coated Yarn‐Type Transpiration‐Driven Electrokinetic Power Generator with High Power Efficiency and Water Stability

**DOI:** 10.1002/advs.202504463

**Published:** 2025-06-25

**Authors:** Hyungsub Yoon, Heebo Ha, Mintaek Hong, Seonghun Lee, Mathis Mortensen Brette, Ji‐Won Jung, Ki Ro Yoon, Ergang Wang, Han Seul Kim, Byungil Hwang, Tae Gwang Yun

**Affiliations:** ^1^ Department of Intelligent Semiconductor Engineering Chung‐Ang University Seoul 06974 Republic of Korea; ^2^ Department of Advanced Materials Engineering Chungbuk National University Chungdae‐ro 1, Seowon‐Gu, Chungcheongbuk‐do Cheongju 28644 Republic of Korea; ^3^ Department of Molecular Science and Technology Ajou University Suwon 16499 Republic of Korea; ^4^ Department of Chemistry and Chemical Engineering Chalmers University of Technology Göteborg 41296 Sweden; ^5^ Department of Materials Science and Engineering Konkuk University Seoul 05029 Republic of Korea; ^6^ Advanced Materials Program Department of Materials Science and Engineering Konkuk University Seoul 05029 Republic of Korea; ^7^ Advanced Textile R&D Department Korea Institute of Industrial Technology (KITECH) 143, Hanggaul‐ro, Sangrok‐gu Ansan Gyeonggi‐do 15588 Republic of Korea; ^8^ Department of Urban, Energy, and Environmental Engineering Chungbuk National University 1 Chungdae‐ro, Seowon‐gu Cheongju Chungbuk 28644 South Korea; ^9^ School of Integrative Engineering Chung‐Ang University Seoul 06974 Republic of Korea

**Keywords:** divinyl sulfone, PEDOT:PSS, silk yarn, water stability, yarn‐type transpiration‐driven electrokinetic power generator

## Abstract

Hydrovoltaic nanogenerators, which harness small quantities of water to generate power, are gaining considerable attention for applications in next‐generation wearable electronics. Conventional hydrovoltaic nanogenerators are constrained by their limited power density and suboptimal long‐term stability. Therefore, a transpiration‐driven electrokinetic power generator (TEPG) based on silk yarn coated with poly(3,4‐ethylenedioxythiophene):poly(styrene sulfonate) (PEDOT:PSS)/divinyl sulfone (DVS) and designed as a wearable hydrovoltaic nanogenerator offering outstanding power generation efficiency and water stability is presented in this study. Given its hydrophilic surface, mechanical durability, and high aspect ratio, silk yarn is used to design a yarn‐based TEPG system to achieve high spatial‐efficiency and maximize volumetric power density. Furthermore, the covalent crosslinking agent, DVS, is introduced to sustain the long‐term, high‐power production efficiency of PEDOT:PSS. The devised yarn‐type TEPG system generates a maximum power of 112 µW cm^−3^ with artificial sweat. A system comprising 25 yarn‐type TEPGs arranged in a series–parallel configuration is implemented utilizing the high spatial‐efficiency of the sewable yarn‐type TEPG. The results demonstrate the potential of wearable hydrovoltaic nanogenerators as next‐generation renewable energy systems for wearable applications.

## Graphical abstract

A PEDOT:PSS‐based yarn‐type transpiration‐driven electrokinetic power generator with a covalently crosslinking agent, divinyl sulfone, is investigated to address the poor water stability of PEDOT:PSS and achieve an exceptional power generation efficiency. The designed device successfully charges commercial capacitors using a biological aqueous solution, demonstrating the potential of the system for green wearable hydrovoltaic nanogenerator systems.

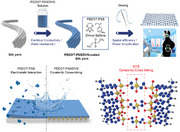

## 1 Introduction

Wearable nanogenerators are gaining prominence as a key technology in the Internet of Things for supplying electrical energy to wearable electronic devices.^[^ ^1^, ^2^, ^3^, ^4^ ^]^ These generators are developed by utilizing small‐scale renewable energy systems, such as piezoelectric and triboelectric effects, which can generate power through mechanical deformation and friction.^[^ ^5^, ^6^, ^7^, ^8^ ^]^ However, these renewable energy systems generate power only briefly (for less than one second), necessitating continuous mechanical deformation and friction for achieving sustainable power generation over longer durations. In addition, piezoelectric and triboelectric energy is produced as high‐frequency alternating current (AC), and therefore, implementing rectifier circuits and conversion systems is necessary to transform the AC into direct current (DC) for a sustained power supply.^[^ ^9^, ^10^ ^]^ Consequently, advancing renewable energy technologies that generate DC power is vital for improving device efficiency and ensuring long‐term stability. Given this context, an innovative concept has emerged for hydrovoltaic nanogenerators that can convert water circulation into DC power. The foundational design of this concept relies on transforming the evaporation of water molecules from a conductive material surface into electrical energy, which can achieve low power efficiency at the nanowatt scale.^[^ ^11^ ^]^

A next‐generation hydrovoltaic nanogenerator has been developed to address this challenge. This nanogenerator directly converts water flow to enhance power generation efficiency. A transpiration‐driven electrokinetic power generator (TEPG), which has emerged as one of promising candidates for green energy harvesting, converting water circulation into electricity across biomimetic structures, includes i) a conductive material that captures ions on its surface, ii) a hydrophilic fibrous structure that facilitates rapid water wicking, and iii) external wiring connections.^[^ ^12^, ^13^ ^]^ Conductive materials with a high surface area physically adsorb ions onto their surfaces to reduce surface energy while electrostatically attracting counter ions to form an electrical double layer. This generates a potential difference between wet and dry regions, thereby enabling the spontaneous movement of charge carriers from the wet region to the dry region. Moreover, the hydrophilic fibrous structure with external wiring connections increases current density by utilizing the movement of water molecules. TEPGs harness small quantities of water to generate electricity continuously, exhibiting notable versatility and sustainability; therefore, they are proposed as a viable alternative for next‐generation wearable power sources.

The efficiency of ion adsorption and the electrical conductivity of the conductive materials are pivotal factors affecting the power generation performance of the TEPG system. A high ion adsorption efficiency enhances charge density in the electrical double layer, whereas superior electrical conductivity facilitates a more substantial flow of electrons, boosting the current output. The integration of conductive materials such as carbon‐based materials,^[^ ^14^, ^15^, ^16^ ^]^ MXene,^[^ ^17^, ^18^, ^19^ ^]^ and conductive polymers^[^ ^20^, ^21^, ^22^, ^23^ ^]^ can significantly optimize the power generation efficiency of TEPGs. Among these, poly(3,4‐ethylenedioxythiophene):poly(styrene sulfonate) (PEDOT:PSS) can maximize power output because of its electrical conductivity and selective cation adsorption properties, enabling cation adsorption within the medium. The negatively charged sulfonate groups of PSS provide ion‐permselectivity, wherein cations are easily absorbed on the surface while electrostatic repulsion effectively impedes the penetration of anions into the PEDOT:PSS matrix.^[^ ^23^, ^24^ ^]^ This results in an additional potential difference that increases power output, which can be attributed to the simultaneous physical and electrostatic adsorptions. Although PEDOT:PSS‐TEPG exhibits effectively improved power efficiency and extended versatility, there are concerns regarding the challenges attributed to the intrinsic physical and chemical properties of PEDOT:PSS. A primary drawback of PEDOT:PSS is its poor morphological stability when exposed to water.^[^ ^25^ ^]^ PEDOT:PSS‐based applications using aqueous solutions suffer from challenges related to its poor stability, which can be attributed to PSS being a hydrophilic polymer.^[^ ^26^, ^27^ ^]^ Therefore, PEDOT:PSS‐TEPG exhibits insufficient long‐term stability and faces challenges in retaining energy generation performance under extreme water exposure conditions, such as during washing. These attributes present a critical drawback, rendering PEDOT:PSS unsuitable for applications in wearable energy devices.

A method that incorporates solvent addition to alter the chemical structure of PEDOT:PSS has been proposed for developing highly water‐resistant PEDOT:PSS. Hwang et al. incorporated ethylene glycol (EG) into PEDOT:PSS and coated it on silk yarn to improve the water resistance.^[^ ^28^ ^]^ The water resistance of the PEDOT:PSS/EG‐coated silk yarn was greatly enhanced without any significant degradation of electrical conductivity, even after 10 cycles of washing with a neutral detergent. Ryan et al. also observed a similar improvement in the water resistance of PEDOT:PSS‐coated silk yarn developed using dimethyl sulfoxide.^[^ ^29^ ^]^ However, these strategies partially remove the PSS groups, which are key components for the adsorption of counter‐ions, thereby degrading the power generation performance of the TEPG system. Therefore, a new strategy that does not remove PSS from PEDOT:PSS is required to develop a highly reliable and efficient TEPG system using PEDOT:PSS.

In this study, we designed a yarn‐type wearable hydrovoltaic nanogenerator using silk yarn to maximize its resistance to water exposure and volumetric power density. Silk yarn exhibits hydrophilic surface properties that facilitate rapid water transport and exceptional mechanical strength among natural yarns, which help maintain its physical properties under mechanical deformation.^[^ ^30^, ^31^ ^]^ In addition, the yarn‐type structure maximizes spatial efficiency because of its high aspect ratio, significantly enhancing volumetric power density. We engineered and uniformly coated a highly water‐resistant PEDOT:PSS material using the silk yarn. Moreover, we promoted the strong crosslinking of PEDOT:PSS by employing a covalent crosslinker agent, divinyl sulfone (DVS), thereby enhancing its water‐resistance capability. We successfully realized a wearable hydrovoltaic nanogenerator with superior power generation performance and reliability using the PEDOT:PSS/DVS‐coated yarn‐type TEPG.

## 2 Results and Discussion

**Figure 1:**
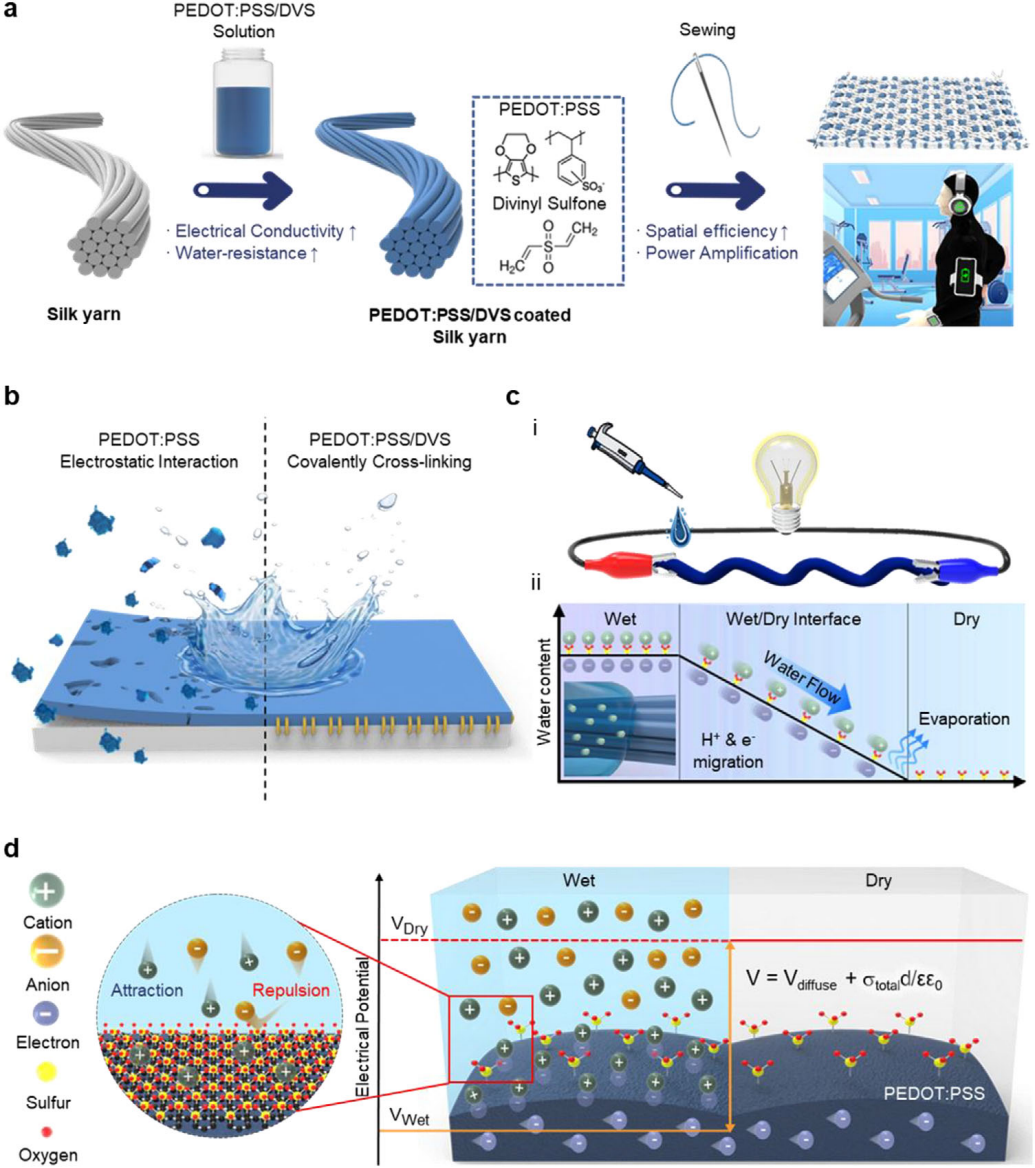
Overview of the PEDOT:PSS/DVS‐coated silk yarn‐type TEPG system. a) Schematic of the fabrication process of the PEDOT:PSS/DVS‐coated silk yarn‐type TEPG system. b) Schematic of the water stability of the PEDOT:PSS‐ and PEDOT:PSS/DVS‐coated silk yarns. c) Schematic of i) the operating method of the yarn‐type TEPG system and ii) the pseudo‐streaming current generation mechanism. d) Schematic of the cation/anion selective interaction with PEDOT:PSS/DVS‐coated silk yarn and its voltage generation mechanism.

**Figure** **1**a shows the overall design of the yarn‐type TEPG‐based wearable hydrovoltaic nanogenerator system. The yarn‐type TEPG was adopted to implement wearable hydrovoltaic nanogenerator systems for the following reasons. First, silk yarn exhibits hydrophilic surface properties crucial for current generations reliant on water wicking. Furthermore, silk yarn demonstrates outstanding wetting properties in polar solvents, enabling the uniform application of conductive material coatings onto the yarns via a dip‐coating process. This simplicity aids industry‐scale mass production, ensuring cost‐ and time‐effectiveness. In addition, the PEDOT:PSS/DVS combination lends itself well to large‐scale processing because of its facile dip‐coating process on hydrophilic silk yarn, thereby ensuring exceptional process efficiency and scalability. Second, silk yarn is suitable for wearable applications because it exhibits remarkable human‐friendly properties and mechanical strength that maintains its physical properties under mechanical deformation. Third, the yarn‐type system exhibits a high volumetric power density. The high aspect ratio of the yarn‐type device results in exceptional spatial efficiency and can amplify the power generation performance through connecting yarn‐type devices in series and parallel. Therefore, we developed a TEPG system tailored for wearable devices, aiming to optimize power generation efficiency using silk yarn as the foundation. We demonstrated the viability of wearable hydrovoltaic nanogenerator technology based on the PEDOT:PSS/DVS‐coated yarn‐type TEPG system and optimized its power generation performance and stability. We fabricated a wearable prototype that integrates 25 optimized PEDOT:PSS/DVS‐coated silk yarns into a waterproof fabric and validated the wearable applicability of the PEDOT:PSS/DVS‐coated yarn‐type TEPG.

PEDOT:PSS/DVS was utilized as a conductive material for yarn‐type TEPG to overcome the poor water stability of conventional PEDOT:PSS (Figure 1b). DVS was used as the covalent crosslinking agent to improve the reliability of PEDOT:PSS when exposed to water. DVS forms strong connections within the PEDOT:PSS matrix through covalent bonding reactions with PEDOT:PSS and cellulose, effectively preventing the delamination of PEDOT:PSS from the silk yarn and enhancing its mechanical robustness.^[^ ^32^, ^33^ ^]^ The basic operation process involves dropping an aqueous solution on one side of yarn‐type TEPGs (Figure 1c–i). In the wet region, cations from the aqueous solution are physically absorbed on the surface of the conductive material with a high surface area to reduce surface energy, forming an electrical double layer with electrons.^[^ ^12^ ^]^ In addition, the negatively charged PSS functional groups within the PEDOT:PSS structures selectively attract cations while repelling anions from the aqueous energy source (Figure 1d).^[^ ^23^ ^]^ This selective attraction enables PEDOT:PSS/DVS‐coated yarn‐type TEPGs to enhance the potential difference by physically adsorbing ions and facilitating selective counter‐ion penetration into the medium. The surface charge density of the PEDOT/DVS‐coated silk yarn is affected by combined charge densities resulting from physical ion absorption and selective attraction by the PSS groups. Therefore, the synergistic effect of physical adsorption and electrostatic interactions maximizes the overall potential difference, enhancing power density. Additionally, the output voltage (*V*_output_) in PEDOT:PSS‐based TEPG system is related with the ion‐permselectivity and its surface charge density, as followed equations and shown in Figure 1d, where *V*_output_, *V*_diffuse_, *σ*_total_, d, ε and ε_o_, are the output voltage, a diffuse layer potential, a total surface charge density, an inner layer thickness and dielectric constant of the medium and free space, respectively. In addition, *σ*_phy_ and *σ*_sulfonate_ represent the surface charge density formed by the physical adsorption of ions and electrostatic attraction of ions by sulfonate groups in PSS. Especially, *σ*_sulfonate_ is proportional to factors of α and N_c_, which are relative cation permselectivity and cation number in the electrolyte.

(1) $$V_{\mathrm{output}}=V_{\mathrm{diffuse}}+\sigma_{\mathrm{total}}d/\varepsilon \varepsilon_{o}$$

(2) $$\sigma_{\mathrm{total}}=\sigma_{\mathrm{pys}}+\sigma_{\mathrm{sulfonate}}$$

(3) $$\sigma_{\mathrm{sulfonate}}\propto \alpha N_{c}$$

To empirically verify the correlation between cation concentration, total surface charge density, and output voltage, we performed a series of experiments by systematically adjusting the NaCl concentration in aqueous electrolyte solutions (0, 0.051, 0.1, 0.2, and 0.4 m) (Figure S1, Supporting Information). Output voltages were measured using PEDOT:PSS/DVS‐coated silk yarns with a fixed electrical resistance of 11 kΩ. The observed *V*_output_ values of 0.039, 0.124, 0.150, 0.205, and 0.255 V, corresponding to the increasing NaCl concentrations, clearly indicate a proportional relationship between *N*_c_ and output voltage. These results provide experimental support for the theoretical model predicting that *V*_output_ increases as a function of both *N*_c_ and *σ*_total_, thereby reinforcing the validity of the proposed theoretical model. Furthermore, as capillary flows of the aqueous solution, the cations from the solution migrate in the direction of the wicking direction of the solution and electrons within PEDOT:PSS/DVS flow along the external circuit, generating the electricity (Figure 1c–ii). The hydrophilic nature of the silk yarn and the presence of an external circuit facilitate the movement of charge carriers through rapid capillary wicking, which enhances the current generation performance. Figure S2 (Supporting Information) depicts the overall schematic process during the power generation of PEDOT:PSS/DVS‐coated yarn‐type TEPG system according to the evaporation time. When aqueous solution is dropped on the PEDOT:PSS/DVS‐coated yarn‐type TEPG, the solution is absorbed into the PEDOT:PSS/DVS‐coated silk yarn and diffused to the dry side by the aqueous solution gradient. During the capillary flow, the power is continuously generated. As time passes and the aqueous source continues to be evaporated, the aqueous solution gradient between wet and dry parts gradually disappears, after which the power generation process is finally ended.

**Figure 2:**
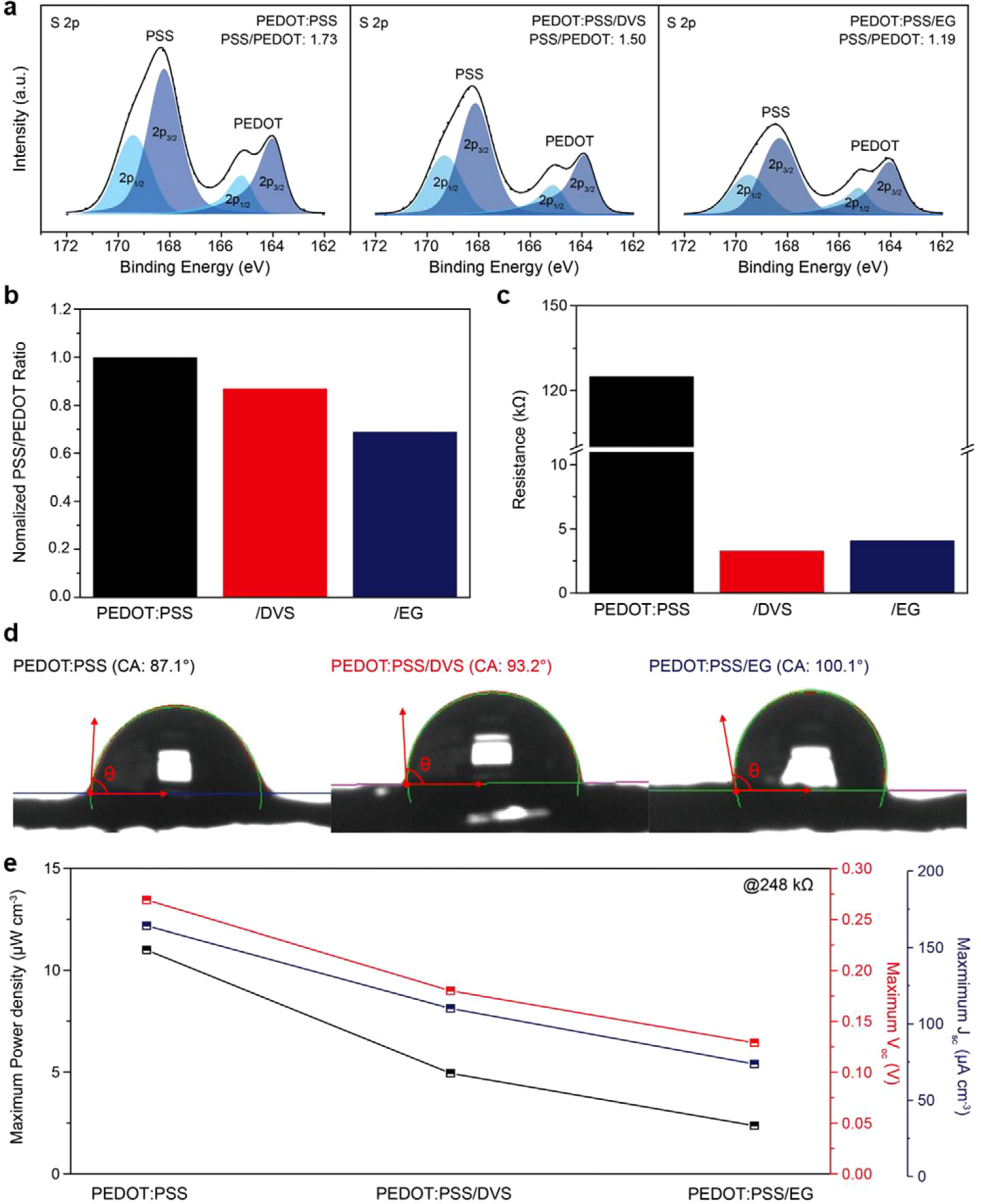
Effect of crosslinking agents on the PEDOT:PSS‐based yarn‐type TEPG systems. a) XPS‐measured S 2p spectra of the PEDOT:PSS‐, PEDOT:PSS/DVS‐, and PEDOT:PSS/EG‐coated silk yarns. b) Comparison of the crosslinking agent‐dependent normalized PSS/PEDOT ratio obtained from the S 2p spectra. c) Comparison of the electrical resistance of PEDOT:PSS‐based yarn‐type TEPG systems. d) Contact angle variation of PEDOT:PSS‐based silk yarns depending on the crosslinking agents obtained by dropping DI water. e) Measured maximum *V*_oc_, *J*_sc_, and volumetric power density values of the PEDOT:PSS‐based yarn‐type TEPG systems obtained by dropping DI water (248 kΩ).

The negative charge of PSS in PEDOT:PSS within the silk yarn selectively permits the permeation of cations from the water resource into the polymer matrix, maximizing energy generation efficiency. However, previous findings indicate that the vulnerability of PEDOT:PSS to water exposure stems from the hydrophilic PSS groups.^[^ ^34^ ^]^ Consequently, recent research trends have shifted toward minimizing PSS content in PEDOT:PSS to address this issue. Although reducing the PSS content in PEDOT:PSS‐based TEPG systems significantly improves reliability by forming a structure more resistant to water exposure, it can also decrease power generation efficiency. Therefore, regulating the PSS content in PEDOT:PSS is essential for optimizing both power generation efficiency and reliability. We developed yarn‐type TEPG systems based on PEDOT:PSS with different crosslinking agents to examine the effect of varying the PSS content on power generation efficiency. We formed a comparison group of crosslinking agents that formed crosslinks through various chemical bonds for establishing the correlation between power generation efficiency and PSS content (**Figure** **2**). We compared the crosslinking agents DVS and EG, which form crosslinks via covalent and hydrogen bonding, respectively.

We analyzed the PSS content on the surfaces of silk yarn coated with PEDOT:PSS, PEDOT:PSS/DVS, and PEDOT:PSS/EG using X‐ray photoelectron spectroscopy (XPS) to investigate the variation in the PSS content associated with different crosslinking agents (Figure 2a). We prepared three samples using identical dip‐coating conditions rather than matching the bulk resistance to maintain consistency in the mass loading of PEDOT:PSS on the silk yarn. Figure 2a shows the S 2p core‐level spectra of PEDOT:PSS‐, PEDOT:PSS/DVS‐, and PEDOT:PSS/EG‐coated silk yarns acquired in the range of 162–172 eV and fitted with symmetric/asymmetric Gaussian–Lorentzian functions. The S 2p peak with a higher binding energy originates from the sulfonate in the PSS groups, whereas the S 2p peak with a lower binding energy originates from the thiophene ring of PEDOT.^[^ ^35^ ^]^ Each S 2p peak derived from PSS and PEDOT was deconvoluted as a spin‐orbit doublet from S 2p_1/2_ and S 2p_3/2_. The ratio of PSS to PEDOT in the PEDOT:PSS‐coated silk yarn was 1.73, whereas that in PEDOT:PSS/DVS and PEDOT:PSS/EG‐coated silk yarns decreased by 1.50 and 1.19, respectively. This result indicates that the secondary doping effect of DVS and EG removed excess PSS from PEDOT:PSS.^[^ ^33^, ^36^ ^]^ Consequently, the PSS and PEDOT ratios of PEDOT:PSS/DVS‐ and PEDOT:PSS/EG‐coated silk yarns were 13% and 31% lower than that of PEDOT:PSS‐coated silk yarn (Figure 2b).

The incorporation of crosslinking agents induced changes in the PSS content, subsequently affecting both the electrical and surface properties of the material. A reduction in the PSS content enhanced electrical conductivity and led to the development of hydrophobic surface characteristics because PSS is an electrically insulating and hydrophilic polymer.^[^ ^25^, ^37^, ^38^ ^]^ The changes in the electrical conductivity and surface properties of PEDOT:PSS‐based silk yarns were verified through electrical resistance and water contact angle measurements (Figure 2c,d). Both crosslinking agents caused secondary doping effects and contributed to the increase in electrical conductivity (Figure 2c). PEDOT:PSS/DVS‐ and PEDOT:PSS/EG‐coated silk yarns demonstrated a 97.4% and 96.7% decrease in the electrical resistance, respectively, compared to that of the PEDOT:PSS‐coated silk yarn. This reduction indicates that both DVS and EG eliminated the electrically insulating excess PSS groups. In short, the incorporation of DVS and EG into the PEDOT:PSS aqueous dispersion facilitates the fabrication of highly conductive structures using a reduced loading of PEDOT:PSS‐based active material, relative to PEDOT:PSS alone. Moreover, the minimum resistance of PEDOT:PSS/DVS‐ and PEDOT:PSS/EG‐coated silk yarns is anticipated to be significantly lower than that of silk yarn coated solely with PEDOT:PSS, underscoring their potential for the development of high‐performance PEDOT:PSS‐based conductive yarns, fibers, textiles, and related electronic applications. In addition, compared to PEDOT:PSS without the crosslinking agent, the surface of PEDOT:PSS with the crosslinking agents exhibited more hydrophobic properties (Figure 2d). Figure 2d shows that PEDOT:PSS‐, PEDOT:PSS/DVS‐, and PEDOT:PSS/EG‐coated silk yarns exhibited deionized (DI) water contact angles of 87.1°, 93.2°, and 100.1°, respectively. The increased contact angles observed for the PEDOT:PSS/DVS‐ and PEDOT:PSS/EG‐coated silk yarns suggested that DVS and EG eliminated hydrophilic excess PSS groups. In addition, PEDOT:PSS/EG‐coated silk yarn showed a higher water contact angle, compared to PEDOT:PSS/DVS‐coated silk yarn, due to the secondary doping effect removing more of the hydrophilic PSS by EG than DVS, which is a consistent result with PSS contents obtained from XPS analysis.

We evaluated the open‐circuit voltage (*V*_oc_), short‐circuit current density (*J*_sc_), and volumetric power density profiles of PEDOT:PSS‐, PEDOT:PSS/DVS‐, and PEDOT:PSS/EG‐coated silk yarns with the same resistance (248 kΩ) to establish the correlation between variations in PSS content induced by the crosslinking agent and power generation performances (Figure 2e; Figure S3a,b, Supporting Information). To enable a consistent evaluation of power generation characteristics under a fixed load of 248 kΩ, samples with resistance deviations confined to within ±1% were exclusively utilized. A higher PSS content ratio was associated with higher *V*_oc_, *J*_sc_, and volumetric power density. Unlike solid‐liquid triboelectric nanogenerators (SL‐TENGs), where hydrophobic surfaces enhance triboelectric performance through effective contact‐separation,^[^ ^39^, ^40^, ^41^, ^42^ ^]^ the hydrovoltaic mechanism of TEPG relies on continuous water‐solid interaction. Therefore, a hydrophilic interface by PSS, hydrophilic polymer, improves ion mobility and water layer stability, resulting in a higher output voltage, as evidenced by the inverse correlation between contact angle and voltage in Figure 2d,e. In the PEDOT:PSS‐coated silk yarn with the highest PSS content, these values were 0.269 V, 164, and 11 µW cm^−3^, respectively, followed by those of PEDOT:PSS/DVS‐, and PEDOT:PSS/EG‐coated silk yarn, which showed the same trend with PSS contents from the XPS analysis. To further verify the importance of the PSS parts for power generation performance of PEDOT:PSS‐based TEPG system, we also addressed the *V*_oc_, *J*_sc_, and volumetric power density of PEDOT:PSS‐, PEDOT:PSS/DVS‐, and PEDOT:PSS/EG‐coated silk yarns with matching the loading amounts on the silk yarns (Figure S4, Supporting Information). When the loading mass of PEDOT:PSS‐, PEDOT:PSS/DVS‐, and PEDOT:PSS/EG on the silk yarns was 0.1 mg, the electrical resistances were 800, 450, and 520 kΩ, respectively. Even though the same loading content of conductive materials was coated on the silk yarns, PEDOT:PSS/DVS‐, and PEDOT:PSS/EG‐coated silk yarns showed lower electrical resistance than PEDOT:PSS‐coated silk yarn, indicating that DVS and EG have a role of secondary doping effect on PEDOT:PSS by reducing PSS contents. PEDOT:PSS‐coated silk yarn at 800 kΩ showed *V*_oc_ of 0.514 V, *J*_sc_ of 77.8 µA cm^−3^, and volumetric power density of 10 µW cm^−3^, respectively. PEDOT:PSS/DVS‐coated silk yarn at 450 kΩ generated 0.217 V, 95.2 µA cm^−3^, and 5.15 µW cm^−3^, respectively, while these values of PEDOT:PSS/EG‐coated silk yarn at 520 kΩ were 0.215 V, 55.8 µA cm^−3^, and 3 µW cm^−3^, respectively. Despite the same loading mass of three PEDOT:PSS‐based silk yarns, PEDOT:PSS‐coated silk yarn with the highest PSS contents showed the highest power generation values, followed by PEDOT:PSS/DVS‐, and PEDOT:PSS/EG‐coated silk yarns. Especially, by comparing PEDOT:PSS/DVS‐, and PEDOT:PSS/EG‐coated silk yarns with the same loading amounts, PEDOT:PSS/DVS‐coated silk yarn showed the higher *V*_oc_ value even with low electrical resistance. These findings demonstrate that the presence of negatively charged PSS groups is crucial for enhancing the power generation performance of PEDOT:PSS‐based TEPGs because the PSS content is directly proportional to the surface charge density, particularly for the adsorption of selective cations. However, PSS groups have water‐induced instability, which affects their long‐term stability. Therefore, given the trade‐off relationship between power generation efficiency and long‐term stability, conditions conducive to optimization must be determined.

**Figure 3:**
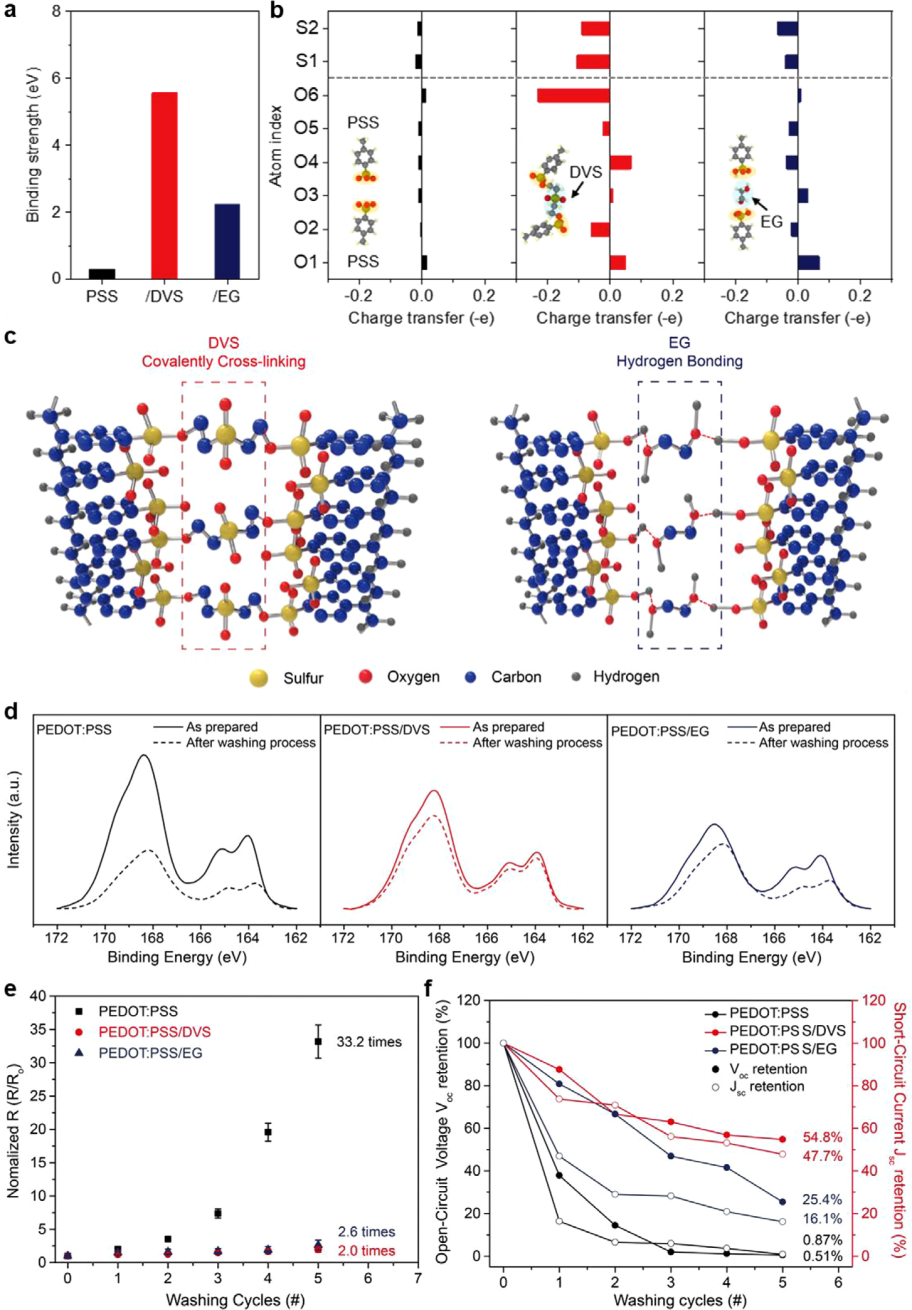
Effect of crosslinking agents on the water resistance of the PEDOT:PSS‐based yarn‐type TEPG system. a) Simulated binding strengths of PSS/PSS, PSS/DVS/PSS, and PSS/EG/PSS models. b) Binding‐induced electron transfer amount projected onto all the oxygen and sulfur atoms inside the SO_3_^−^ groups. Yellow and blue shaded regions indicate SO_3_^−^ groups and crosslinking agents, respectively. c) Crosslinking mechanisms of DVS (covalently crosslinking) and EG (hydrogen bonding) in PEDOT:PSS. d) Comparison of the XPS‐measured S 2p spectra of the PEDOT:PSS‐, PEDOT:PSS/DVS‐, and PEDOT:PSS/EG‐coated silk yarns before and after washing. e) Normalized resistance change in PEDOT:PSS‐, PEDOT:PSS/DVS‐, and PEDOT:PSS/EG‐coated silk yarns as a function of the number of washing cycles. f) Measured *V*_oc_, and *J*_sc_ retention of the PEDOT:PSS‐based yarn‐type TEPG systems after multiple washing cycles.

Although a decrease in power production efficiency was observed because of the decline in PSS content, PEDOT:PSS with crosslinking agents is used because of its exceptional stability in aqueous environments. The improved water resistance was demonstrated by evaluating moisture exposure stability (**Figure** **3**). Prior to the stability assessment, we conducted density functional theory (DFT) simulations to evaluate how crosslinking agents modify the theoretical binding strength between PEDOT:PSS backbones via covalent and hydrogen bonding (Figure 3a). The binding strength was determined based on the computed binding energy, with its definition provided in the Experimental Section. To simplify the model, we adopted PSS/PSS, PSS/DVS/PSS, and PSS/EG/PSS configurations (inset images of Figure 3b). The extracted binding strengths were 0.30, 5.57, and 2.25 eV for PSS/PSS, PSS/DVS/PSS, and PSS/EG/PSS, respectively. As designed, the weak van der Waals interaction in PSS/PSS was significantly reinforced by the introduction of crosslinking agents, with covalent bonding in DVS yielding the highest increase (5.57 eV) and hydrogen bonding in EG providing a moderate enhancement (2.25 eV). The binding‐induced charge transfer in all sulfur and oxygen atoms within the SO_3_^−^ groups of PSS units is shown in Figure 3b and Figure S5 (Supporting Information). Weak van der Waals bonding in PSS/PSS resulted in minimal charge transfer (<0.016 **e^−^**), while DVS induced a tenfold increase (up to 0.106 **e^−^** loss for sulfur and 0.23 **e^−^** loss for oxygen) by reflecting its covalent bonding nature. The introduction of EG led to an intermediate transfer (up to 0.064 **e^−^** loss for sulfur and 0.067 **e^−^** gain for oxygen) by indicating its hydrogen bonding interactions, which were higher than in PSS/PSS but lower than in PSS/DVS/PSS. These simulation results highlight the critical role of crosslinking agents in strengthening the interactions between adjacent PSS backbones. In particular, covalent bonding agents provide a much stronger enhancement through extensive interatomic bonding and charge transfer, while hydrogen bonding agents offer a more moderate effect.

Fourier transform infrared (FT‐IR) measurements of the PEDOT:PSS, PEDOT:PSS/DVS, and PEDOT:PSS/EG films were conducted to analyze the crosslinking mechanism based on the crosslinking agents (Figure S6, Supporting Information). New peaks appeared at 3100–3000 cm^−1^ (C═CH_2_ stretching vibration) and 1312 cm^−1^ (OSO scissoring) after adding DVS in PEDOT:PSS; these peaks are the signature peaks of covalently bonded PEDOT:PSS and DVS.^[^ ^32^, ^43^ ^]^ Compared to the PEDOT:PSS film, the peak intensity at 1128 cm^−1^ (C─O stretching) increased dramatically, indicating a crosslinking reaction of DVS with PEDOT:PSS. Unlike PEDOT:PSS and PEDOT:PSS/DVS films, the FT‐IR spectrum of PEDOT:PSS/EG film exhibited a broad peak at 3300 cm^−1^ (O─H stretching band), which is ascribed by the hydroxyl group from EG, leading to hydrogen bonding with PEDOT:PSS. In addition, peaks at 2925 and 2863 cm^−1^ were ascribed to the C─H asymmetric and symmetric stretching vibration from EG, respectively.^[^ ^44^, ^45^ ^]^ The increase in peak intensity at 2362–2021 cm^−1^ (S─H stretching) indicated the intermolecular hydrogen bonding between PEDOT:PSS and EG.^[^ ^45^, ^46^ ^]^ These results confirmed that crosslinking reactions of DVS or EG with PEDOT:PSS were successfully achieved. The possible crosslinking mechanisms of PEDOT:PSS/DVS and PEDOT:PSS/EG are depicted in Figure 3c. The sulfonate groups of PSS were combined with DVS and EG through covalent and hydrogen bonding, respectively, which enhanced water resistance compared to that of PEDOT:PSS.

Based on the theoretical insights into stability obtained from DFT simulations, the durability of the actual yarn‐type device was further investigated. To evaluate its suitability for wearable applications, the water resistance was examined by simulating an extreme water‐exposure environment, similar to an actual washing procedure. The simulated stir‐washing procedure was performed by immersing the samples in a commercial neutral detergent solution diluted at 0.33 mL per 250 mL of water, which corresponds to the manufacturer's recommended concentration. The solution was agitated at 150 rpm for 5 min at 25 °C. The detergent used is free of enzymes, phosphates, and optical brighteners, and its formulation is comparable to ISO reference detergents (Type 3 or 6). Following the washing step, the samples were rinsed with 250 mL of DI water under the same agitation and temperature conditions to remove any residual detergent. Subsequently, the washed and rinsed samples were dried in an oven at 60 °C for 30 min after each washing and drying cycle. The water resistance was evaluated by monitoring changes in the loading mass of PEDOT:PSS and the variations in the XPS intensity on the yarn surface (Figure 3d; Figures S7 and S8, Supporting Information). Figure 3d shows the XPS spectra of the PEDOT:PSS‐coated yarn samples before and after washing. A notable reduction was observed in the XPS intensity of the PEDOT:PSS upon rapid water exposure, which contrasts with the intensity observed for the initially prepared PEDOT:PSS‐coated silk yarn. Thus, PEDOT:PSS readily delaminates and separates from the surface of the silk yarn because of the presence of hydrophilic PSS groups and weak electrostatic interactions in the PEDOT:PSS‐coated silk yarn. Conversely, the DVS and EG crosslinking agents exhibited robust water resistance by forming strong chemical bonds between polymer backbones. DVS effectively suppressed the delamination of PEDOT:PSS by forming strong atomic‐level covalent bonds. In addition, the enhanced binding force between PEDOT:PSS and the silk yarn generated by the Oxa–Michael addition reaction of DVS contributed to the remarkable water resistance of the PEDOT:PSS/DVS‐coated silk yarn compared to those of other samples.^[^ ^33^, ^47^, ^48^ ^]^ The field‐emission scanning electron microscopy (FE‐SEM) images of the PEDOT:PSS‐based silk yarns after washing revealed that individual pristine silk fibers were clearly visible in the case of PEDOT:PSS‐based silk yarns after exposure to a rapid water environment. However, PEDOT:PSS/DVS‐ and PEDOT:PSS/EG‐coated silk yarns prevented the delamination of PEDOT:PSS from the surface of the silk yarns (Figure S7a–c, Supporting Information). PEDOT:PSS/DVS‐coated silk yarns retained more PEDOT:PSS on the silk yarn than that with PEDOT:PSS/EG‐coated silk yarns, which implies that DVS can better address the poor water stability of PEDOT:PSS. Furthermore, as shown in the real images, most of PEDOT:PSS was eliminated from the silk yarn after washing, while PEDOT:PSS/DVS‐ and PEDOT:PSS/EG‐coated silk yarns were maintained on the silk yarns (Figure S8, Supporting Information).

Changes in the electrical resistance, water contact angles, and power production efficiency of the device were measured to verify that the electrical properties and power generation efficiency of PEDOT:PSS remained intact after the washing procedure (Figure 3e,f; Figures S9, S10, and S11, Supporting Information). Figure 3e shows that the normalized electrical resistance of the PEDOT:PSS‐coated silk yarn doubled after the first washing cycle and increased by 33.2 ± 2.5 times after five cycles compared with the initial value. In contrast, the PEDOT:PSS/DVS‐ and PEDOT:PSS/EG‐coated silk yarns exhibited excellent water‐resistance characteristics, with only 2 ± 0.4 and 2.6 ± 0.75 times increase in electrical resistance compared with the initial resistance after five washing cycles. The PEDOT:PSS/DVS‐coated silk yarn demonstrated ≈1660% and ≈130% improvements in electrical‐resistance stability after five washing cycles compared to those of the PEDOT:PSS‐ and PEDOT:PSS/EG‐coated silk yarns, respectively. The changes in water contact angles of three different PEDOT:PSS‐based silk yarns depending on the washing cycles were shown in Figures S9, S10, and S11 (Supporting Information). Contact angles were recorded before washing, and after the 1st, 3rd, and 5th washing cycles. For PEDOT:PSS‐coated silk yarn, the water contact angle decreased significantly from 87.1° (before washing) to 80.1° (after 1st washing) and 23.0° (after 3rd washing). After the 5th cycle, the PEDOT:PSS coating was severely degraded, and contact angle could no longer be measured, indicating delamination and poor washing stability of pristine PEDOT:PSS (Figure S9a,b, Supporting Information). In contrast, PEDOT:PSS/DVS‐coated sample exhibited stable wettability, with contact angles of 93.2°, 95.2°, 90.6°, and 96.4° before and after the 1st, 3rd, and 5th washes, respectively (Figure S10a,b, Supporting Information). This demonstrates that DVS crosslinking effectively enhances water resistance by covalently bonding PSS chains. For PEDOT:PSS/EG‐coated samples, the contact angles were 100.1°, 98.2°, 95.2°, and 90.6° across the same cycles (Figure S11a,b, Supporting Information). While EG also improved wash durability by promoting hydrogen bonding between PSS chains, the effect was less pronounced than DVS. These results indicate that both DVS and EG improve the waterproof characteristics of PEDOT:PSS‐coated yarns, but covalent crosslinking by DVS offers superior stability compared to the non‐covalent hydrogen bonding of EG. These outstanding water‐resistant characteristics suggest exceptional power generation efficiency even under extreme conditions, signifying high feasibility for wearable applications. Specifically, the PEDOT:PSS‐, PEDOT:PSS/DVS‐, and PEDOT:PSS/EG‐coated silk yarns were subjected to over five washing cycles under controlled conditions, with voltage and current generation measured after each cycle (Figure 3f; Figure S12, Supporting Information). In practice, the PEDOT:PSS/DVS‐coated yarn‐type TEPG showed the most minor degradation in the output voltage (54.8%) and current (47.7%), respectively, despite five times washing, demonstrating robust retention of device performance, compared to PEDOT:PSS‐ (0.51% for voltage retention, and 0.87% for current retention), PEDOT:PSS/EG‐coated silk yarns (25.4% for voltage retention, and 16.1% for current retention). These results suggest the successful improvement of yarn‐type TEPG with exceptional water resistance, compared to PEDOT:PSS alone. Additionally, to evaluate the stability of power generation under repeated operation, the retention of *V*_oc_ and *J*_sc_ was investigated for PEDOT:PSS‐, PEDOT:PSS/DVS‐, and PEDOT:PSS/EG‐coated silk yarns with a resistance of 248 kΩ by applying five successive drops of DI water (Figure S13, Supporting Information). After five cycles, the PEDOT:PSS/DVS‐coated yarn‐type TEPG retained 79.5%, 85.8%, and 68.3% of the initial *V*_oc_, *J*_sc_, and volumetric power density, respectively. This was followed by the PEDOT:PSS/EG‐coated silk yarn, which exhibited retention rates of 59.7% for *V*_oc_, 40.5% for *J*_sc_, and 24.2% for volumetric power density. In contrast, the PEDOT:PSS‐coated yarn‐type TEPG showed significantly lower retention values of 19.3%, 23%, and 4.42% for *V*_oc_, *J*_sc_, and volumetric power density, respectively. These results demonstrate that the incorporation of DVS into PEDOT:PSS significantly enhances the water stability, confirming the effectiveness of our crosslinking agent strategy to improve bonding strength. The radar chart assessing electrical conductivity, hydrophilicity, power generation, and water resistance revealed that the PEDOT:PSS/DVS‐coated silk yarn‐based TEPG demonstrated superior suitability for wearable hydrovoltaic nanogenerators compared to those of PEDOT:PSS and PEDOT:PSS/EG‐coated silk yarn‐based TEPGs (Figure S14, Supporting Information).

**Figure 4:**
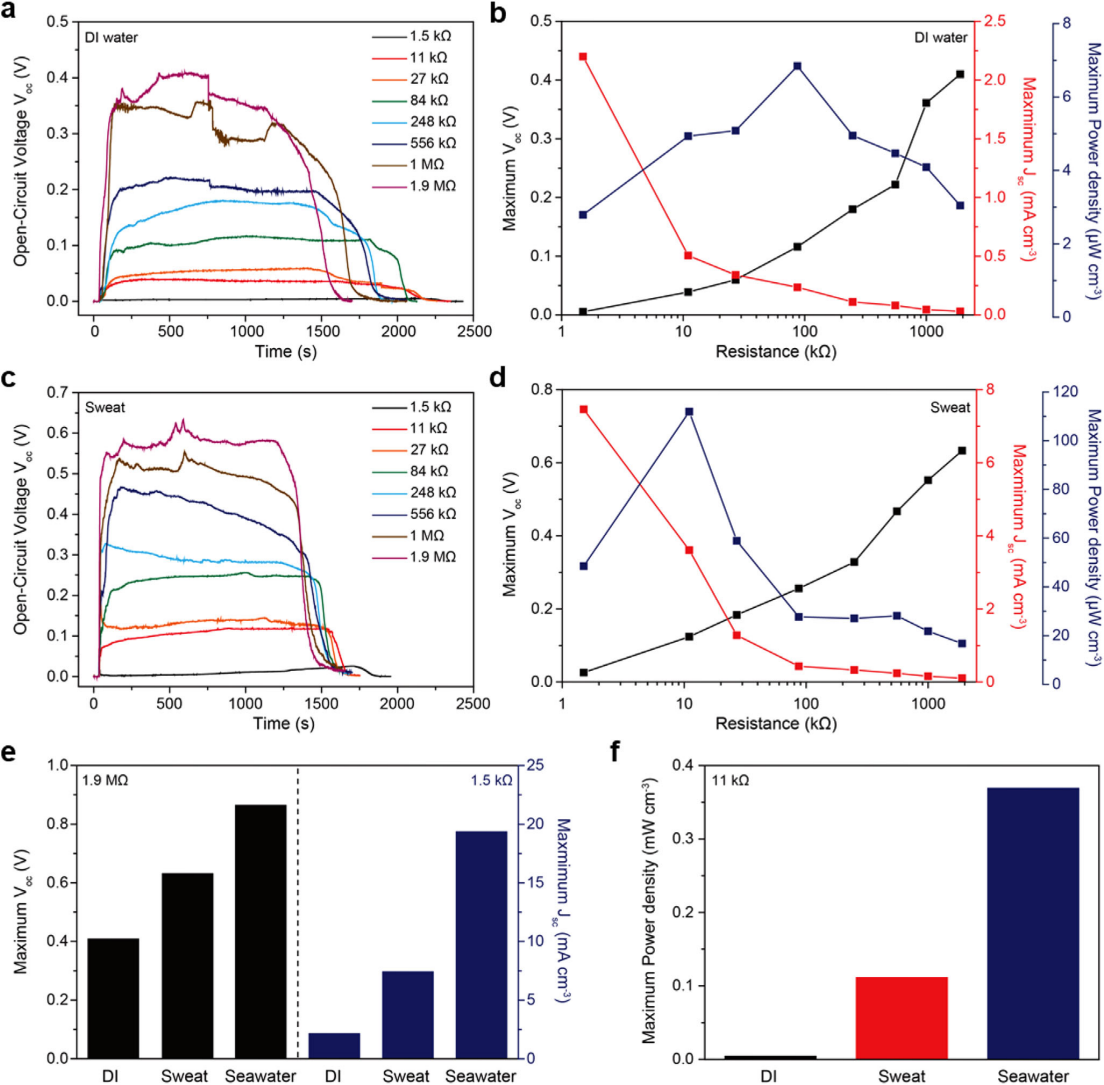
Power generation performance of the PEDOT:PSS/DVS‐coated yarn‐type TEPG system. a) Measured *V*_oc_ profiles obtained by dropping DI water on PEDOT:PSS/DVS‐coated yarn‐type TEPG systems with various resistances. b) Comparison of the measured maximum *V*_oc_, *J*_sc_, and volumetric power density values of the PEDOT:PSS/DVS‐coated yarn‐type TEPG system with various resistances obtained by dropping DI water. c) Measured *V*_oc_ profiles obtained by dropping a sweat salt solution on PEDOT:PSS/DVS‐coated yarn‐type TEPG systems with various resistances. d) Comparison of the measured maximum *V*_oc_, *J*_sc_, and volumetric power density values of the PEDOT:PSS/DVS‐coated yarn‐type TEPG systems with various resistances obtained by dropping a sweat salt solution. e) Measured maximum *V*_oc_ (1.9 MΩ) and maximum *J*_sc_ (1.5 kΩ) obtained by dropping various types of energy resources on the PEDOT:PSS/DVS‐coated yarn‐type TEPG system. f) Comparison of the maximum power density values of the PEDOT:PSS/DVS‐coated yarn‐type TEPG system (11 kΩ) with various types of energy resources.

The measured *V*_oc_ and *J*_sc_ profiles of the PEDOT:PSS/DVS‐coated yarn‐type TEPG were measured based on changes in the resistance of the device to maximize its power generation (**Figure** **4**). The electrical resistance of the PEDOT:PSS/DVS‐coated silk yarn‐based devices (ranging from 1.5 kΩ to 1.9 MΩ) was systematically tuned by controlling the loading amount of active material, which was achieved by immersing the yarn in aqueous dispersions of varying concentrations. As the resistance increased, *V*_oc_ exhibited a gradual enhancement, while *J*_sc_ correspondingly decreased. This inverse relationship underscores the importance of optimizing device resistance to achieve maximum power output. Accordingly, a comprehensive characterization of the voltage and current responses across the resistance range was conducted, as presented in Figure 4a–d. Based on the measured *V*_oc_ and *I*_sc_, the power density was calculated to determine the resistance condition that yields the highest power generation efficiency. The performance of the yarn‐type TEPG devices with different resistances was comparatively evaluated by applying a fixed volume (5 µL) of DI water (Figure 4a,b; Figure S15, Supporting Information). The highest *J*_sc_ of 2.2 mA cm^−3^ was attained at the lowest resistance (1.5 kΩ), whereas the maximum generated *V*_oc_ (0.41 V) was obtained at the highest resistance (1.9 MΩ). However, the volumetric power densities at 1.5 kΩ and 1.9 MΩ were only 2.79 and 3.04 µW cm^−3^, respectively, which can be attributed to the low open‐circuit voltage at 1.5 kΩ (0.00507 V) and short‐circuit current density at 1.9 MΩ (29.7 µA cm^−3^). The highest volumetric power density (6.84 µA cm^−3^) was obtained at an optimized resistance of 87 kΩ, along with a *V*_oc_ of 0.116 V and *J*_sc_ of 236 µA cm^−3^. Therefore, optimizing the resistance is crucial for maximizing the power generation performance. Additionally, for the long‐term stability evaluation, the power generation performance of the PEDOT:PSS/DVS‐coated yarn‐type TEPG device, exhibiting an optimized electrical resistance of ≈87 kΩ, was monitored over a 5‐day period through repeated DI water application (Figure S16, Supporting Information). Following each measurement, the samples were stored under controlled ambient conditions (temperature: 25 °C, relative humidity (RH): 50%). After 5 days, the device retained 96.1% of its initial *V*_oc_ and 84.3% of its initial *J*_sc_ (Figure S16a–c, Supporting Information). The volumetric power density retention was measured to be ≈81% of the initial value (Figure S16d, Supporting Information), indicating sustained device performance under prolonged operation. Furthermore, since the performance of the TEPG system is intrinsically linked to ambient environmental factors, notably relative humidity and temperature, due to their direct influence on the evaporation kinetics of the aqueous electrolyte. To elucidate the relationship between evaporation rate and both the temporal stability and magnitude of power output, we systematically investigated the device's electrical response under controlled temperature and relative humidity conditions, employing a consistent aqueous solution volume of 5 µL (Figures S17 and S18, Supporting Information). Specifically, *V*_oc_ and *J*_sc_ measurements were conducted on an 11 kΩ PEDOT:PSS/DVS‐coated silk yarn‐based TEPG, utilizing sweat salt solution under varied humidity and temperature environments. Experiments were carried out in a sealed chamber where relative humidity was controlled between 30% and 80% at a fixed temperature (Figure S17, Supporting Information). The measured output voltage and current showed negligible variation across the humidity range within the margin of experimental error, suggesting that humidity exerts little effect on the amount of electricity generated. Nonetheless, increasing humidity notably slowed the evaporation rate, which in turn significantly prolonged the duration of power generation, extending beyond 10 000 s under conditions of high humidity. A comparable pattern emerged when temperature varied: although the output magnitude remained relatively stable, elevated temperatures accelerated evaporation, causing a considerable decrease in the length of electricity production (Figure S18, Supporting Information). Together, these results indicate that evaporation dynamics, regulated by environmental parameters like humidity and temperature, play a critical role in determining the operational lifespan and energy output of the TEPG system. These findings align with prior research, further confirming that although humidity and temperature impact the longevity of power generation, they exert negligible effect on the peak output voltage and current.^[^ ^12^, ^23^ ^]^

The power generation of PEDOT:PSS/DVS‐coated yarn‐type TEPG was demonstrated using simulated sweat from the human body as an energy resource to demonstrate its potential for wearable applications (Figure 4c,d; Figure S19, Supporting Information). The *V*_oc_ and *J*_sc_ profiles were measured by dropping a 0.051 M NaCl salt solution, which is the same NaCl concentration as a normal sweat.^[^ ^49^ ^]^ Similar to power generation using DI water, at 1.5 kΩ, we observed the highest short‐circuit current density and lowest open‐circuit voltage, recorded at 7.46 mA cm^−3^ and 0.026 V, respectively, which resulted in a volumetric power density of 48.49 µW cm^−3^. Furthermore, at 1.9 MΩ, the PEDOT:PSS/DVS‐coated silk yarn‐based TEPG generated the highest open‐circuit voltage of 0.633 V and the lowest short‐circuit current density of 106 µA cm^−3^, resulting in a volumetric power density of 16.77 µW cm^−3^. At an optimized resistance of 11 kΩ, the highest volumetric power density (112 µW cm^−3^) was achieved, accompanied by an open‐circuit voltage of 0.124 V and a short‐circuit current density of 3.61 mA cm^−3^. The volumetric power density exhibited a 22.7‐fold enhancement compared to that achieved using DI water at 11 kΩ (4.933 µW cm^−3^). This enhancement can be attributed to the contribution of the Na^+^ cations, which increase the charge concentration in the solution, thereby enhancing the total surface charge density and improving power generation.^[^ ^50^, ^51^ ^]^ In addition, salt solutions have higher electrical conductivity than pure DI water, resulting in a decrease in resistance during solution wicking.^[^ ^52^, ^53^ ^]^ Moreover, wearable energy systems are required to sustain stable power generation performance under mechanical deformation. To investigate the applicability of the proposed system for wearable devices, the power generation characteristics of the PEDOT:PSS/DVS‐coated silk yarn‐based TEPG were evaluated under a bending radius of 2 cm. As presented in Figure S20 (Supporting Information), the output voltage and current retained over 95% of their initial values despite the applied mechanical bending. These results demonstrate that the developed coated silk yarn‐based TEPG exhibits excellent mechanical robustness and considerable potential as a reliable energy harvesting platform for wearable applications.

Based on the advantageous role of salt, we further increased the salt concentration up to 0.4 m NaCl (NaCl concentration of seawater) to amplify power generation (Figure 4e; Figures S21 and S22, Supporting Information).^[^ ^23^ ^]^ The open‐circuit voltage at 1.9 MΩ increased from 0.41 V (DI water) and 0.633 V (sweat salt solution) to 0.866 V (seawater salt solution) with an increase in the salt concentration from DI water to that of a seawater salt solution. Moreover, by dropping the seawater salt solution, the short‐circuit current density at 1.5 kΩ reached 19.4 mA cm^−3^, which is 8.8 times and 2.6 times higher than that obtained by dropping DI water (2.2 mA cm^−3^) and sweat salt solution (7.46 mA cm^−3^). Subsequently, we measured the power generation of PEDOT:PSS/DVS‐coated yarn‐type TEPG at an optimized resistance (11 kΩ) when the best power generation performance was achieved with the sweat salt solution (Figure 4f; Figure S23, Supporting Information). The volumetric power density reached 0.37 mW cm^−3^ when the seawater salt solution was dropped; the open‐circuit voltage and short‐circuit current density were 0.255 V and 5.8 mA cm^−3^, respectively. The volumetric power density generated by the seawater salt solution was 75‐ and 3.3‐fold higher than those achieved by the DI water and sweat salt solution, respectively. Compared to previous research using PEDOT:PSS‐coated cotton fabric, where a volumetric power density of 40.7 µW cm^−3^ was achieved using the same seawater salt solution (0.4 m NaCl),^[^ ^23^ ^]^ the proposed PEDOT:PSS/DVS‐coated yarn‐type TEPG generated a substantial volumetric power density, which was 9.1 times higher. This indicates that a yarn‐type TEPG with a high aspect ratio is beneficial for achieving an outstanding volumetric power density. Although a higher salt concentration can further improve the power generation performance up to a certain critical point,^[^ ^16^, ^17^, ^19^ ^]^ we only used the seawater concentration (0.4 m NaCl) because overconcentration indicates an artificial water resource and is unsuitable for practical hydrovoltaic nanogenerator systems. These outstanding results confirm that the PEDOT:PSS/DVS‐coated silk yarn‐based TEPG is a suitable design for wearable hydrovoltaic nanogenerator systems because it exhibits both excellent water resistance and power generation performance, even when using a biological solution (sweat salt solution).

**Figure 5:**
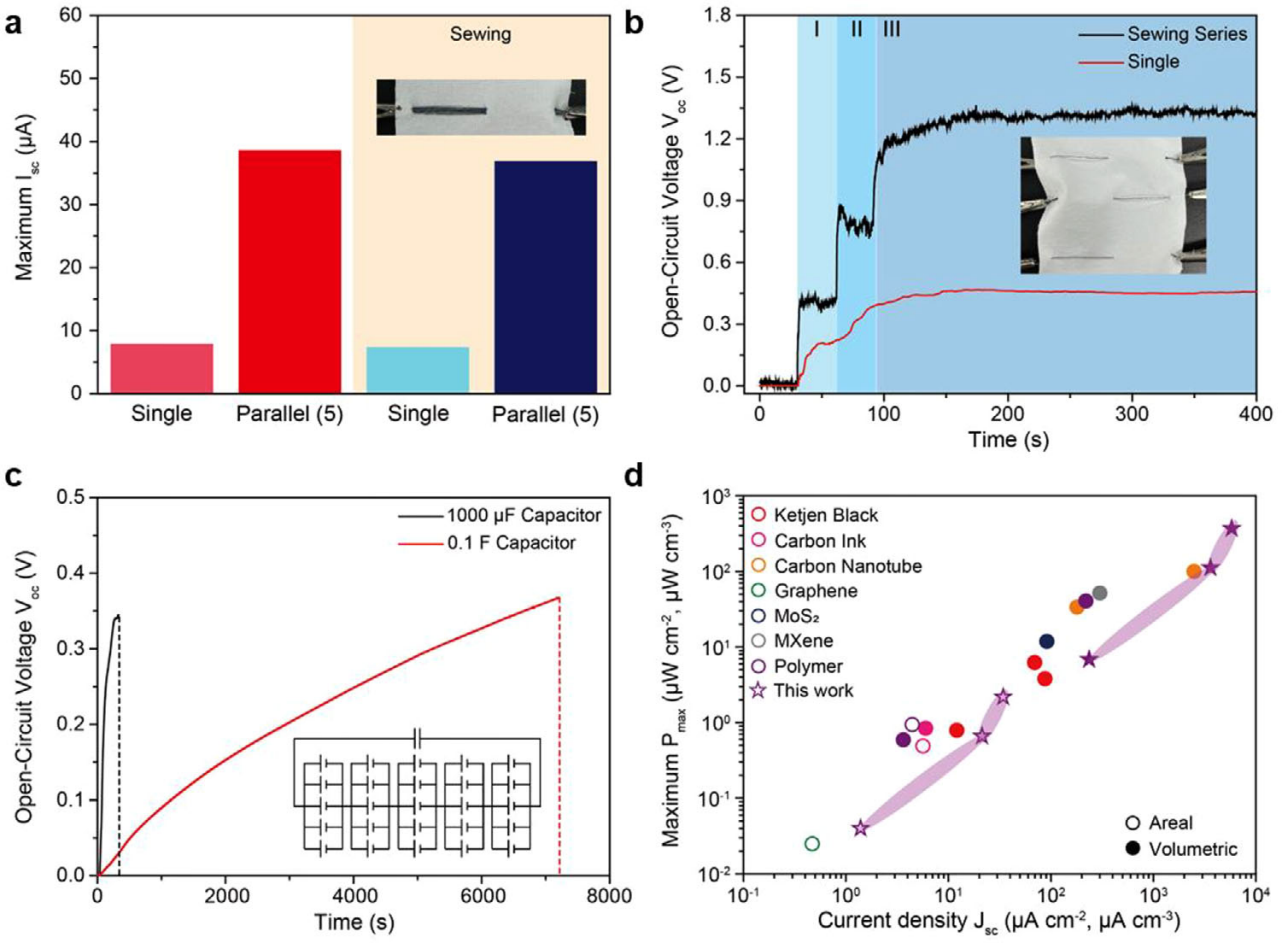
Scalability and demonstration of the PEDOT:PSS/DVS‐coated yarn‐type TEPG system. a) Measured maximum *I*_sc_ of multiple PEDOT:PSS/DVS‐coated yarn‐type TEPGs (11 kΩ) connected in parallel obtained by dropping a sweat salt solution (orange colored region: after sewing the PEDOT:PSS/DVS‐coated yarn‐type TEPGs on waterproof textile). Inset: Photographic image of sewing five PEDOT:PSS/DVS‐coated yarn‐type TEPGs connected in parallel on a waterproof textile. b) Measured stepwise *V*_oc_ profiles of three individually sewn PEDOT:PSS/DVS‐coated yarn‐type TEPGs (1 MΩ) connected in series, obtained by dropping a sweat salt solution. Inset: Photographic image of sewing three PEDOT:PSS/DVS‐coated yarn‐type TEPGs in series on a waterproof textile. c) Charging curve of the commercial capacitor (1000 µF and 0.1 F) obtained by sewing 25 PEDOT:PSS/DVS‐coated yarn‐type TEPGs and dropping a sweat salt solution. Inset: Circuit diagram. d) Performance comparison between various TEPG systems with different conductive materials.

For scalability and demonstration, we connected multiple PEDOT:PSS/DVS‐coated yarn‐type TEPGs in parallel and series (**Figure** **5**). A single PEDOT:PSS/DVS‐coated yarn‐type TEPG wetted with a sweat salt solution generated a short‐circuit current (*I*_sc_) of 7.9 µA, while that of five yarn‐type TEPGs connected in parallel was amplified to *I*_sc_ of 38.6 µA (Figure 5a; Figure S24, Supporting Information). Subsequently, we demonstrated the practicality of PEDOT:PSS/DVS‐coated yarn‐type TEPGs as wearable hydrovoltaic nanogenerator systems by sewing them on a flexible waterproof textile in parallel. A waterproof textile was used to exclude the textile effect on wetting and power generation performance. Sewing a single PEDOT:PSS/DVS‐coated yarn‐type TEPG on the textile generated the *I*_sc_ of 7.37 µA, while sewing five yarn‐type TEPGs connected in parallel obtained the *I*_sc_ of 36.4 µA. These results confirm that sewing PEDOT:PSS/DVS‐coated yarn‐type TEPGs on a waterproof textile did not affect much its power generation performance (Figure 5a; Figure S24, Supporting Information). In addition, we sewed three individual PEDOT:PSS/DVS‐coated yarn‐type TEPGs onto the textile in series to amplify voltage generation, which resulted in an open‐circuit voltage of 1.37 V after dropping the sweat salt concentration (Figure 5b). Moreover, the power generation of the PEDOT:PSS/DVS‐coated yarn‐type TEPG was significantly amplified with outstanding spatial efficiency because of the high aspect ratio of silk yarn, indicating that the yarn‐type TEPG demonstrated high applicability to wearable textile‐based electronics (inset images of Figure 5a,b). Finally, we sewed 25 PEDOT:PSS/DVS‐coated yarn‐type TEPGs onto the textile using five series and five parallel connections to charge commercial capacitors with 1000 µF and 0.1 F (Figure 5c). The 1000 µF capacitor could be charged to 0.35 V within only 330 s, while the 0.1 F capacitor could be charged to 0.37 V within 7200 s by dropping a sweat salt solution. Thus, the PEDOT:PSS/DVS‐coated silk yarn‐type TEPG demonstrates significant potential as a wearable hydrovoltaic nanogenerator system with outstanding power generation performance and excellent washing stability for application in self‐powered wearable electronics.

## 3 Conclusion

We fabricated a highly reliable PEDOT:PSS/DVS‐coated silk yarn‐based TEPG with exceptional power generation efficiency for wearable hydrovoltaic nanogenerators. The exceptional performance can be attributed to the synergistic effect of each material. The silk‐based yarn‐type TEPG system provided an optimized spatial efficiency that significantly enhanced volumetric power density. We also confirmed that the addition of DVS to PEDOT:PSS helped achieve long‐term stability and high power production efficiency because of the enhanced water resistance afforded by the strong covalent bonding while sustaining the PSS functional group. The PEDOT:PSS/DVS‐coated silk yarn‐based TEPG achieved unprecedented current and power generation outputs when fueled with DI water (263 µA cm^−3^ and 6.84 µW cm^−3^), sweat salt solution (3610 µA cm^−3^ and 112 µW cm^−3^), and seawater salt solution (5800 µA cm^−3^ and 0.37 mW cm^−3^), surpassing previous TEPG systems employing different types of conductive materials (Figure 5d; Table S1, Supporting Information). In addition, the electrical resistance of the PEDOT:PSS‐DVS‐coated silk yarn increased by only 2 ± 0.4 times of its initial resistance after five washes, whereas those of the PEDOT:PSS‐ and PEDOT:PSS‐EG‐coated silk yarns demonstrated 33.2 ± 2.5 and 2.6 ± 0.75 times higher initial resistances, respectively. In addition, the retention of open‐circuit voltage and short‐circuit current density after five times washing cycles decreased in the following order: PEDOT:PSS/DVS (54.8% and 47.7%), PEDOT:PSS/EG (25.4% and 16.1%), and PEDOT:PSS (0.51% and 0.87%), respectively. A total of 25 yarn‐type TEPGs were integrated into a series–parallel system that charged commercial energy storage systems using a sweat salt solution, demonstrating the potential of this green wearable hydrovoltaic nanogenerator system. Importantly, the yarn‐type TEPG operates without the necessity of continuous liquid supply, relying instead on the establishment and maintenance of a wet‐dry asymmetry along the yarn axis. This wet‐dry gradient can be intrinsically maintained in wearable applications, where the inner surface of the textile absorbs perspiration from the skin, while the outer surface remains exposed to ambient air, generating a stable moisture gradient. Given the ionic conductivity of sweat, it functions as a biologically compatible electrolyte, providing a sustainable energy source for continuous TEPG operation. Beyond sweat, alternative water sources such as rainwater or environmental humidity offer viable energy inputs, particularly in outdoor scenarios. To ensure device reliability and consistent performance under such variable conditions, the yarns are encapsulated with moisture‐permeable protective layers, allowing stable operation even in wet or high‐humidity environments. The silk‐based yarn substrate affords superior mechanical flexibility and compatibility with diverse textile integration strategies. Furthermore, the modular architecture of the system facilitates series and parallel configurations of multiple TEPG units, enabling adjustable electrical outputs suitable for capacitor charging or powering low‐energy wearable electronics. This modular and flexible design underscores the scalability and practical applicability of the proposed yarn‐type TEPG platform for real‐world energy harvesting applications. We anticipate that our proposed washable TEPG system with outstanding power generation performance will offer attractive insights into next‐generation renewable energy systems.

## 4 Experimental Section

### Fabrication of the PEDOT:PSS‐Based Silk Yarns

Bombyx mori silk yarn (average diameter: ≈236 µm, Aurora Silk) was prewashed for 30 min by stirring at 200 rpm in a mixed solution of DI water (500 mL), neutral detergent (2.02 mL), and ammonia (2.02 mL) at 40 °C to remove any contamination. The PEDOT:PSS‐coated silk yarn was fabricated by immersing the prewashed silk yarn in a PEDOT:PSS aqueous dispersion (1.1 wt%, Sigma Aldrich). The PEDOT:PSS‐coated silk yarn was dried in an oven at 60 °C for 30 min. To make PEDOT:PSS/DVS‐ or PEDOT:PSS/EG‐coated silk yarns, 5 vol% of DVS (>96.0%, Sejin CI Co. Ltd.) or EG (99.5%, Samchun Chem.) were applied to the PEDOT:PSS aqueous dispersion and followed the same process with the PEDOT:PSS‐coated silk yarn. The loading mass was controlled by immersing the silk yarn into various concentrations of each PEDOT:PSS aqueous dispersion to vary the electrical resistance of the PEDOT:PSS‐based silk yarns.

### Electrical Characterization of the PEDOT:PSS‐Coated Silk Yarn‐Based TEPGs

*V*_oc_ and *I*_sc_ of the PEDOT:PSS‐coated silk yarn‐based TEPGs were independently measured using a Keithley 6517A electrometer under controlled conditions of 25 °C and RH 50%. For electrical characterization, a 7 cm‐long PEDOT:PSS‐coated silk yarn‐based TEPG was interfaced with two external steel electrodes. An aqueous solution (5 µL) was applied at one end, 5 cm away from the other side of the device. Therefore, when the *J*_sc_ and volumetric power density of yarn‐type TEPGs were calculated, the effective length of the yarn was 5 cm. The short‐circuit current density was calculated by dividing the short‐circuit current by the volume of the yarn‐type TEPG. The volumetric power density was obtained by calculating the *V*_oc_ and *J*_sc_. The volumetric power density is obtained using

(4) $$\begin{array}{c} Volumetric\;power\;density\;\left(P\right)=\frac{V_{\mathrm{oc}}\times J_{\mathrm{sc}}}{4} \end{array}$$

The PEDOT:PSS‐based yarn‐type TEPGs were placed in a beaker filled with 250 mL of DI water and 0.33 mL of a commercial neutral silk detergent (Aekyung), which is the recommended usage amount specified on the product, to evaluate their stability in water. The composition of the neutral detergent primarily includes water and various surfactants, such as anionic surfactants, including sodium alkylbenzene sulfonate and myristic acid, as well as non‐ionic surfactants, including alkoxylated alcohol and ethoxylated alcohol. The simulated washing process was performed by stirring the solution at 150 rpm for 5 min at 25 °C using a magnetic stirrer. The washed PEDOT:PSS‐based yarn‐type TEPGs were rinsed with plain DI water under the same condition as the washing process to eliminate the detergent residue. The variations in electrical resistance and retention of *V*_oc_ and *J*_sc_ were measured after drying the samples in an oven at 60 °C for 30 min following each washing and drying cycle.

### Characterization

XPS (K‐alpha+, Thermo Fisher Scientific, USA) was performed using an Al‐Kα radiation source to elucidate the PSS contents on the surface of the PEDOT:PSS‐coated silk yarns according to the crosslinking agents. In addition, XPS measurements were performed to investigate the effect of the crosslinking agents on the washing process by comparing the samples before and after washing. The surface morphology of the PEDOT:PSS‐based silk yarns before and after washing was characterized using FE‐SEM (Carl Zeiss AG, Germany). The PEDOT:PSS, PEDOT:PSS/DVS, and PEDOT:PSS/EG films were analyzed using a FT‐IR spectrometer (Bruker, Alpha II, USA) to explore the bonding mechanism between PEDOT:PSS and the crosslinking agents. The contact angles of the PEDOT:PSS silk yarns were measured with DI water using a drop shape analyzer (DSA‐100, KRUSS, Germany).

### Electronic Structure Calculations

DFT calculations were performed using the SIESTA (Spanish Initiative for Electronic Simulations with Thousands of Atoms) package.^[^ ^54^ ^]^ A double‐ζ plus‐polarization basis set was employed, and core electrons were described using Troullier–Martins‐type norm‐conserving pseudopotentials.^[^ ^55^ ^]^ The exchange‐correlation potential was treated with the Perdew–Burke–Ernzerhof (PBE) functional within the generalized gradient approximation (GGA).^[^ ^56^ ^]^ To efficiently deal with Van der Waals interactions, Grimme's D3 corrections were adopted.^[^ ^57^ ^]^ To prevent artificial interactions between periodic images in the PSS/PSS, PSS/DVS/PSS, and PSS/EG/PSS models, a vacuum layer of at least 20 Å was introduced in all *x*, *y*, and *z* directions. The geometries were relaxed until the total energy and atomic forces converged to 10^−4^ eV and 0.01 eV Å^−1^, respectively. To effectively quantify the binding strength, it is defined as the negative of the binding energy:

(5) $$\begin{array}{c} \;S_{\mathrm{binding}}=-\;E_{\mathrm{binding}}=-\left(E_{\mathrm{PSS}/\mathrm{crosslinker}/\mathrm{PSS}}+2\times E_{\mathrm{PSS}}-E_{\mathrm{crosslinker}}\right) \end{array}$$

where *E*_PSS/crosslinker/PSS_, *E*_PSS_, and *E*_crosslinker_ are the total energies of the crosslinked system, single PSS unit, and crosslinking agent, respectively. By this definition, a larger positive binding strength indicates a stronger interaction, making it a more intuitive measure of binding affinity.

## Conflict of Interest

The authors declare no conflict of interest.

## Supporting information



Supporting Information

## Data Availability

The data that support the findings of this study are available from the corresponding author upon reasonable request.
