# Supplementary material for: Wearable PEDOT:PSS/DVS‐Coated Yarn‐Type Transpiration‐Driven Electrokinetic Power Generator with High Power Efficiency and Water Stability
Source: Adv Sci (Weinh). 2025 Jun 25;12(35):e04463. doi: 10.1002/advs.202504463 (PMC12462974; doi:10.1002/advs.202504463)
Supplement: Supplementary file 1 — Supporting Information [file ADVS-12-e04463-s001.docx]

Wearable PEDOT:PSS/DVS-coated Yarn-type Transpiration-driven Electrokinetic Power Generator with High Power Efficiency and Water Stability

Hyungsub Yoon^†^, Heebo Ha^†^, Mintaek Hong^†^, Seonghun Lee, Mathis Mortensen Brette, Ji-Won Jung, Ki Ro Yoon, Ergang Wang^*^, Han Seul Kim^*^, Byungil Hwang^*^, and Tae Gwang Yun^*^

H. Yoon, H. Ha

Department of Intelligent Semiconductor Engineering, Chung-Ang University, Seoul 06974, Republic of Korea

B. Hwang

School of Integrative Engineering, Chung-Ang University, Seoul 06974, Republic of Korea

E-mail: bihwang@cau.ac.kr

M. Hong, H. S. Kim

Department of Advanced Materials Engineering, Chungbuk National University, Chungdae-

ro 1, Seowon-Gu, Cheongju, Chungcheongbuk-do 28644, Republic of Korea

E-mail: hanseul.kim@chungbuk.ac.kr

H. S. Kim

Department of Urban, Energy, and Environmental Engineering, Chungbuk National University, 1 Chungdae-ro, Seowon-gu, Cheongju, Chungbuk, 28644 Korea

S. Lee, T. G. Yun

Department of Molecular Science and Technology, Ajou University, Suwon 16499, Republic of Korea

E-mail: ytk0402@ajou.ac.kr

M. Brette, E. Wang

Department of Chemistry and Chemical Engineering, Chalmers University of Technology, Göteborg, 41296 Sweden

E-mail: ergang@chalmers.se

J.W. Jung

Department of Materials Science and Engineering, Konkuk University, Seoul 05029, Republic of Korea, Advanced Materials Program, Department of Materials Science and Engineering, Konkuk University, Seoul 05029, Republic of Korea.

K. R. Yoon

Advanced Textile R&D Department, Korea Institute of Industrial Technology (KITECH), 143, Hanggaul-ro, Sangrok-gu, Ansan, Gyeonggi-do, 15588, Republic of Korea

^†^These authors contributed equally to this work.

**Table of Contents**

**Figure S1.** The output voltage of PEDOT:PSS/DVS-coated silk yarn-based TEPG system with 11 kΩ obtained by dropping 5 μl of different NaCl concentration in aqueous solution. (a) Measured V_oc_ profiles. (b) Comparison of the maximum V_oc_ values.

**Figure S2.** Evaporation-Time-Dependent Power Generation Mechanism of PEDOT:PSS/DVS-Coated Silk Yarn-Based TEPG.

**Figure S3.** Measured (a) V_oc_ and (b) J_sc_ profiles obtained by dropping 5 μl of DI water on PEDOT:PSS-, PEDOT:PSS/DVS-, and PEDOT:PSS/EG-coated yarn-type TEPG systems (248 kΩ).

**Figure S4.** Measured (a) V_oc_ and (b) J_sc_ profiles obtained by dropping 5 μl of DI water on PEDOT:PSS-, PEDOT:PSS/DVS-, and PEDOT:PSS/EG-coated yarn-type TEPG systems with same loading mass (0.1 mg). Comparison of the maximum (c) V_oc_ and J_sc_ values, and (d) power density value of PEDOT:PSS-, PEDOT:PSS/DVS-, and PEDOT:PSS/EG-coated yarn-type TEPG systems with same loading mass (0.1 mg).

**Figure S5.** Net electron count on oxygen and sulfur atoms (a–c) before and (d–f) after the formation of (a,d) PSS/PSS, (b,e) PSS/DVS/PSS, and (c,f) PSS/EG/PSS bonds.

**Figure S6.** FTIR spectra of PEDOT:PSS, PEDOT:PSS/DVS, and PEDOT:PSS/EG films.

**Figure S7.** Surface morphologies of PEDOT:PSS-, PEDOT:PSS/DVS-, and PEDOT:PSS/EG-coated silk yarns before and after washing.

**Figure S8.** Photographic images of PEDOT:PSS-, PEDOT:PSS/DVS-, and PEDOT:PSS/EG- coated silk yarns before and after washing.

**Figure S9.** (a) Digital images and (b) comparison of contact angle variation of PEDOT:PSS-coated silk yarns depending on the washing cycles obtained by dropping DI water

**Figure S10.** (a) Digital images and (b) comparison of contact angle variation of PEDOT:PSS/DVS-coated silk yarns depending on the washing cycles obtained by dropping DI water

**Figure S11.** (a) Digital images and (b) comparison of contact angle variation of PEDOT:PSS/EG-coated silk yarns depending on the washing cycles obtained by dropping DI water

**Figure S12.** Washing stability measurements of yarn-type TEPG systems obtained by dropping 5 μl of DI water after multiple washing cycles. Measured (a, c, and e) V_oc_, and (b, d, and f) J_sc_ profiles of PEDOT:PSS-, PEDOT:PSS/DVS-, and PEDOT:PSS/EG-coated yarn-type TEPG systems, respectively.

**Figure S13.** The stability measurement of PEDOT:PSS-, PEDOT:PSS/DVS-, and PEDOT:PSS/EG-coated yarn-type TEPG system at 248 kΩ obtained by repeatedly 5 times dropping DI water. Measured (a, c and e) V_oc_, and (b, d, and f) J_sc_ profiles by repeatedly 5 times dropping DI water. (g) V_oc_ and J_sc_ and (h) maximum power density retention as function of the drop cycles.

**Figure S14.** Radar chart of PEDOT:PSS-based yarn-type TEPG systems for evaluating the electrical conductivity, hydrophilicity, power generation, and water resistance.

**Figure S15.** Measured J_sc_ profiles obtained by dropping 5 μl of DI water on PEDOT:PSS/DVS-coated yarn-type TEPG systems with various resistances.

**Figure S16.** The stability measurement of PEDOT:PSS/DVS-coated yarn-type TEPG system at 87 kΩ obtained by dropping DI water during 5 days. Measured (a) V_oc_, and (b) J_sc_ profiles during 5 days. (c) V_oc_ and J_sc_ and (d) maximum power density retention as function of the days.

**Figure S17.** (a) Schematic illustration of the system for the experiment depending on the relative humidity. (b) Digital image of the system at RH 30 % (Top) and RH 80 % (Bottom). Measured (c) V_oc_ profiles and (d) J_sc_ profiles of PEDOT:PSS/DVS-coated yarn-type TEPG system (11 kΩ) by dropping sweat salt solution at RH 30, 50, and 80 % with same temperature 25 °C.

**Figure S18.** (a) Schematic illustration of the system for the experiment depending on the temperature. (b) Digital image of the system at 50 °C and RH 50 %. Measured (c) V_oc_ profiles and (d) J_sc_ profiles of PEDOT:PSS/DVS-coated yarn-type TEPG system (11 kΩ) by dropping sweat salt solution at 25 and 50 °C with same RH 50 %.

**Figure S19.** Measured J_sc_ profiles obtained by dropping 5 μl of the sweat salt solution on PEDOT:PSS/DVS-coated yarn-type TEPG systems with various resistances.

**Figure S20.** (a) Digital image of the experimental system for measuring power generation performance under deformation. Measured (b) V_oc_ profiles and (c) J_sc_ profiles of PEDOT:PSS/DVS-coated yarn-type TEPG system (11 kΩ) by dropping sweat salt solution. (d) Comparison of maximum V_oc_ and J_sc_ values with and without bending.

**Figure S21.** Measured V_oc_ profiles of PEDOT:PSS/DVS-coated yarn-type TEPG systems with various types of energy resources (1.9 MΩ).

**Figure S22.** Measured J_sc_ profiles of PEDOT:PSS/DVS-coated yarn-type TEPG systems with various types of energy resources (1.5 kΩ).

**Figure S23.** Measured (a) V_oc_ and (b) J_sc_ profiles of PEDOT:PSS/DVS-coated yarn-type TEPG systems with various types of energy resources (11 kΩ).

**Figure S24.** Measured I_sc_ profiles obtained by dropping 5 μl of the sweat salt solution on multiple PEDOT:PSS/DVS-coated yarn-type TEPG systems (11 kΩ) before and after sewing on waterproof textile in parallel.

**Table S1.** Comparison of power generation performance of various TEPG systems with different conductive absorbents.

**Supplementary references**


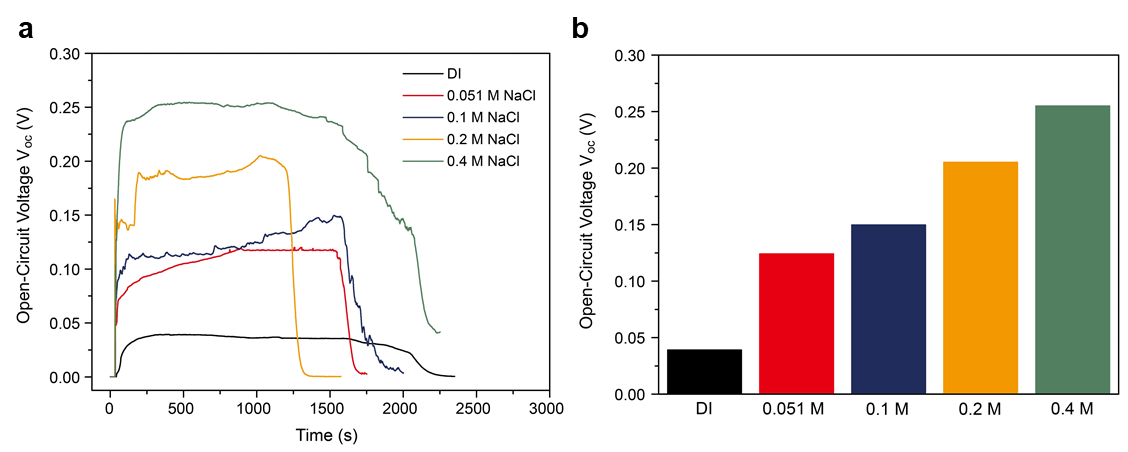


**Figure S1.** The output voltage of PEDOT:PSS/DVS-coated silk yarn-based TEPG system with 11 kΩ obtained by dropping 5 μl of different NaCl concentration in aqueous solution. (a) Measured V_oc_ profiles. (b) Comparison of the maximum V_oc_ values.

**Figure S2.** Evaporation-Time-Dependent Power Generation Mechanism of PEDOT:PSS/DVS-Coated Silk Yarn-Based TEPG.

**Figure S3.** Measured (a) V_oc_ and (b) J_sc_ profiles obtained by dropping 5 μl of DI water on PEDOT:PSS-, PEDOT:PSS/DVS-, and PEDOT:PSS/EG-coated yarn-type TEPG systems (248 kΩ).

**Figure S4.** Measured (a) V_oc_ and (b) J_sc_ profiles obtained by dropping 5 μl of DI water on PEDOT:PSS-, PEDOT:PSS/DVS-, and PEDOT:PSS/EG-coated yarn-type TEPG systems with same loading mass (0.1 mg). Comparison of the maximum (c) V_oc_ and J_sc_ values, and (d) power density value of PEDOT:PSS-, PEDOT:PSS/DVS-, and PEDOT:PSS/EG-coated yarn-type TEPG systems with same loading mass (0.1 mg).

**Figure S5.** Net electron count on oxygen and sulfur atoms (a–c) before and (d–f) after the formation of (a,d) PSS/PSS, (b,e) PSS/DVS/PSS, and (c,f) PSS/EG/PSS bonds.


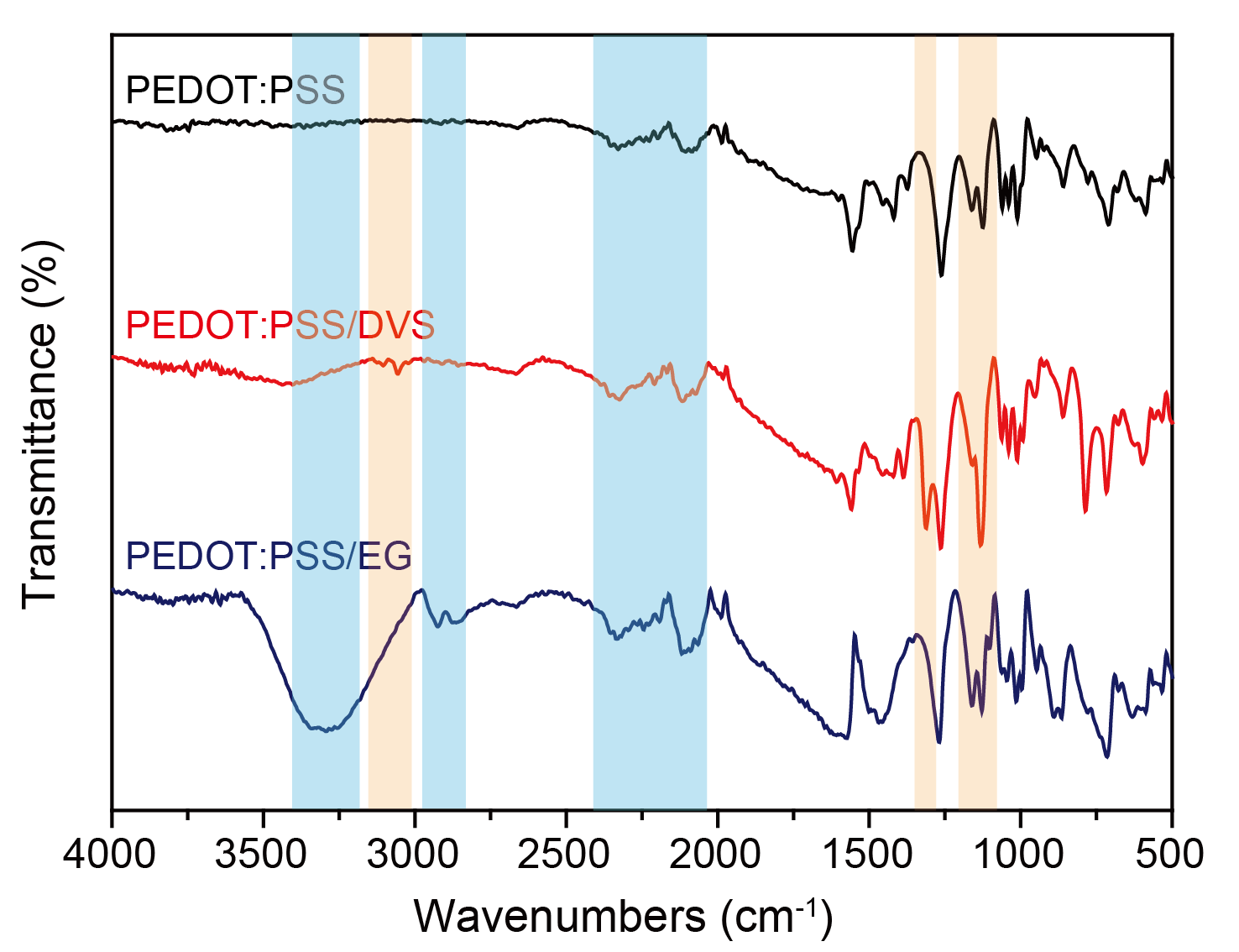


**Figure S6.** FTIR spectra of PEDOT:PSS, PEDOT:PSS/DVS, and PEDOT:PSS/EG films.


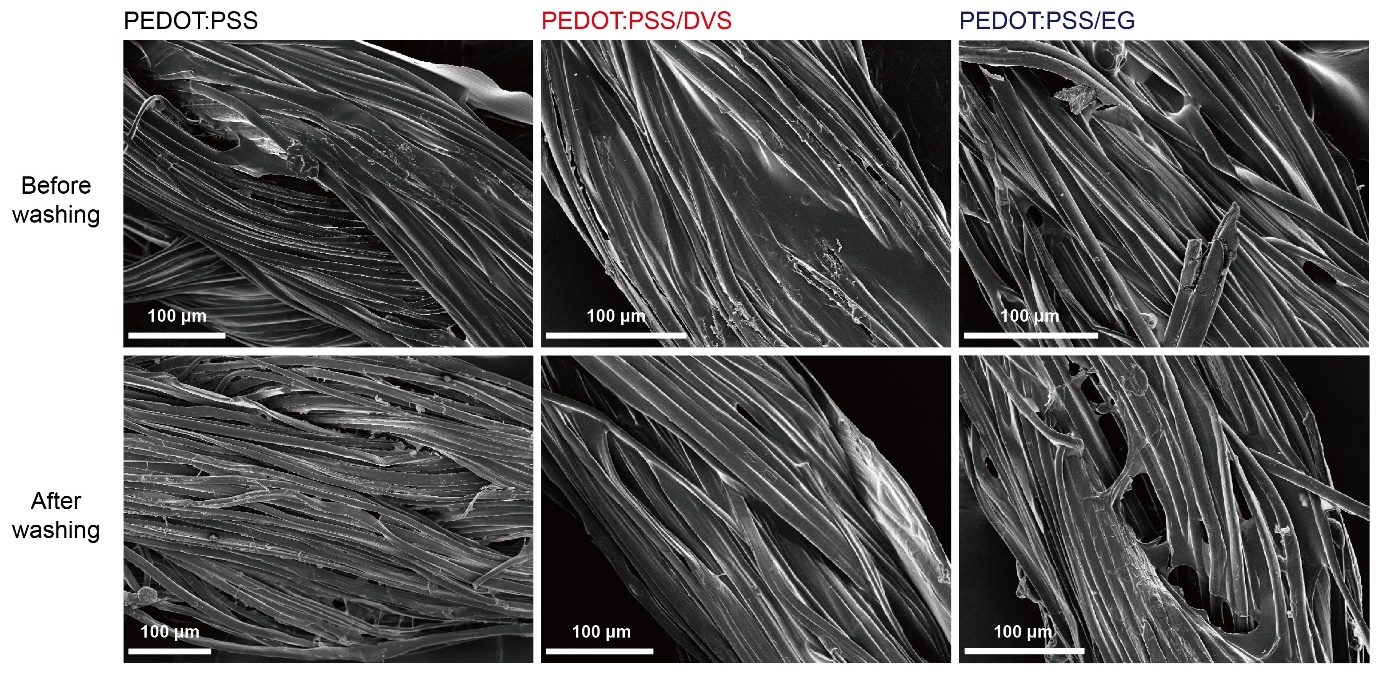


**Figure S7.** Surface morphologies of PEDOT:PSS-, PEDOT:PSS/DVS-, and PEDOT:PSS/EG-coated silk yarns before and after washing.


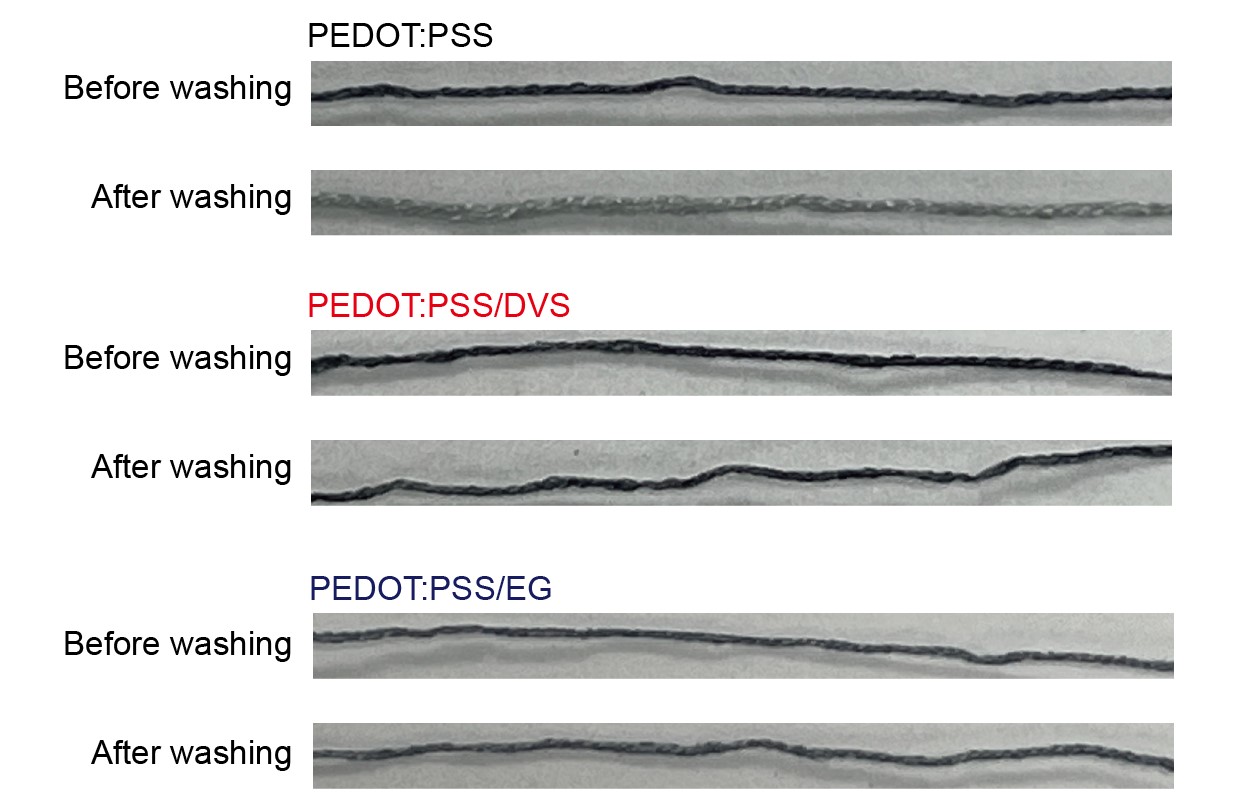


**Figure S8**. Photographic images of PEDOT:PSS-, PEDOT:PSS/DVS-, and PEDOT:PSS/EG- coated silk yarns before and after washing.

**Figure S9.** (a) Digital images and (b) comparison of contact angle variation of PEDOT:PSS-coated silk yarns depending on the washing cycles obtained by dropping DI water.

**Figure S10.** (a) Digital images and (b) comparison of contact angle variation of PEDOT:PSS/DVS-coated silk yarns depending on the washing cycles obtained by dropping DI water.

**Figure S11.** (a) Digital images and (b) comparison of contact angle variation of PEDOT:PSS/EG-coated silk yarns depending on the washing cycles obtained by dropping DI water.


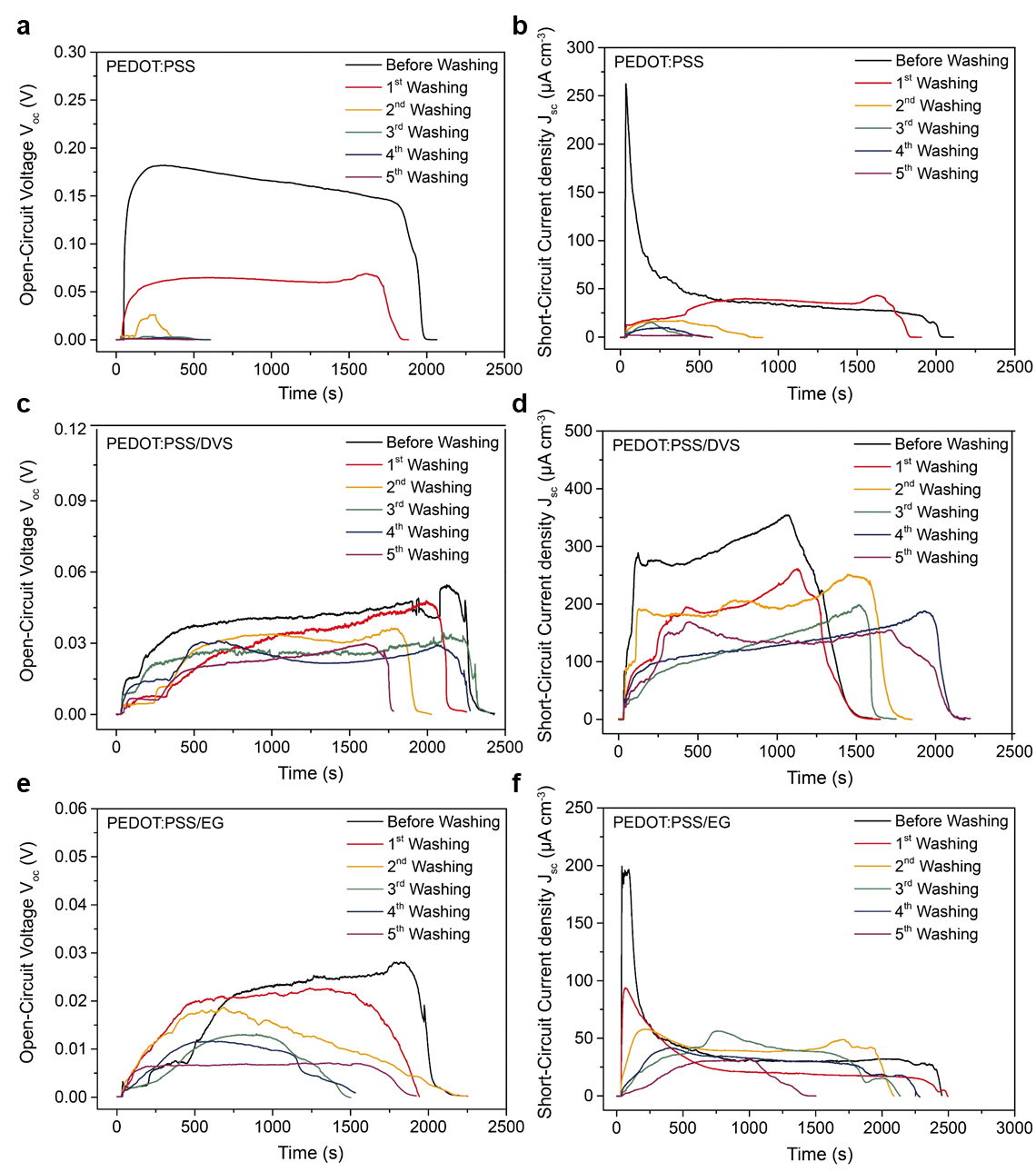


**Figure S12.** Washing stability measurements of yarn-type TEPG systems obtained by dropping 5 μl of DI water after multiple washing cycles. Measured (a, c, and e) V_oc_, and (b, d, and f) J_sc_ profiles of PEDOT:PSS-, PEDOT:PSS/DVS-, and PEDOT:PSS/EG-coated yarn-type TEPG systems, respectively.

**Figure S13.** The stability measurement of PEDOT:PSS-, PEDOT:PSS/DVS-, and PEDOT:PSS/EG-coated yarn-type TEPG system at 248 kΩ obtained by repeatedly 5 times dropping DI water. Measured (a, c and e) V_oc_, and (b, d, and f) J_sc_ profiles by repeatedly 5 times dropping DI water. (g) V_oc_ and J_sc_ and (h) maximum power density retention as function of the drop cycles.

**Figure S14.** Radar chart of PEDOT:PSS-based yarn-type TEPG systems for evaluating the electrical conductivity, hydrophilicity, power generation, and water resistance.


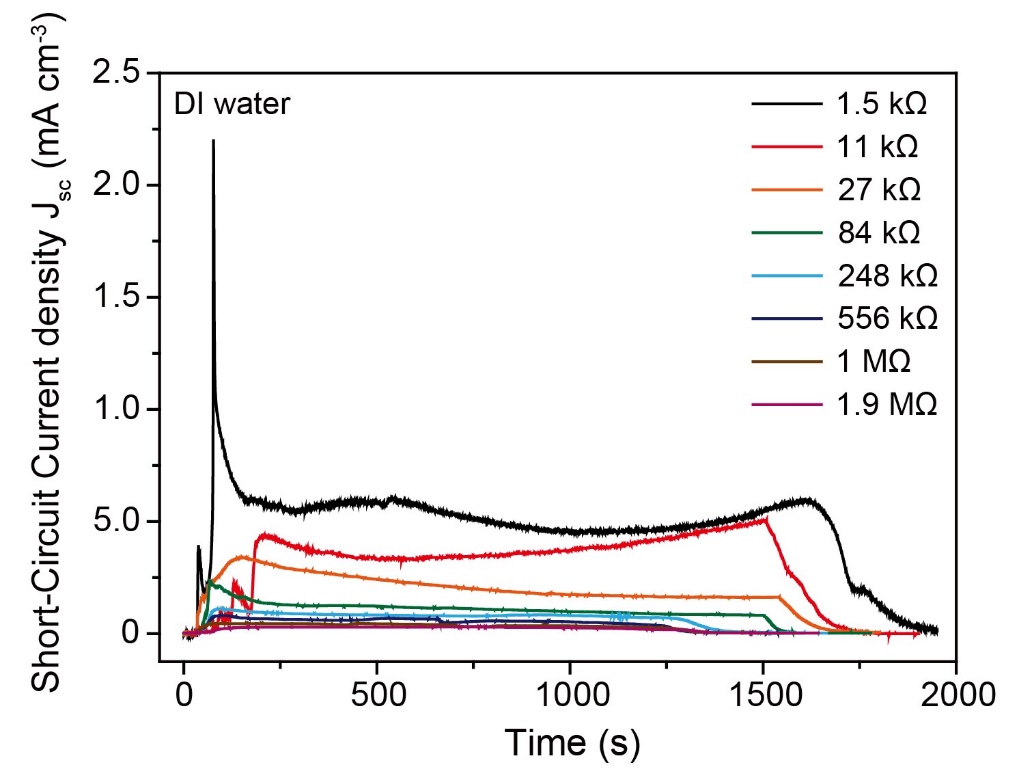


**Figure S15.** Measured J_sc_ profiles obtained by dropping 5 μl of DI water on PEDOT:PSS/DVS-coated yarn-type TEPG systems with various resistances.

**Figure S16.** The stability measurement of PEDOT:PSS/DVS-coated yarn-type TEPG system at 87 kΩ obtained by dropping DI water during 5 days. Measured (a) V_oc_, and (b) J_sc_ profiles during 5 days. (c) V_oc_ and J_sc_ and (d) maximum power density retention as function of the days.

**Figure S17.** (a) Schematic illustration of the system for the experiment depending on the relative humidity. (b) Digital image of the system at RH 30 % (Top) and RH 80 % (Bottom). Measured (c) V_oc_ profiles and (d) J_sc_ profiles of PEDOT:PSS/DVS-coated yarn-type TEPG system (11 kΩ) by dropping sweat salt solution at RH 30, 50, and 80 % with same temperature 25 °C.

**Figure S18.** (a) Schematic illustration of the system for the experiment depending on the temperature. (b) Digital image of the system at 50 °C and RH 50 %. Measured (c) V_oc_ profiles and (d) J_sc_ profiles of PEDOT:PSS/DVS-coated yarn-type TEPG system (11 kΩ) by dropping sweat salt solution at 25 and 50 °C with same RH 50 %.


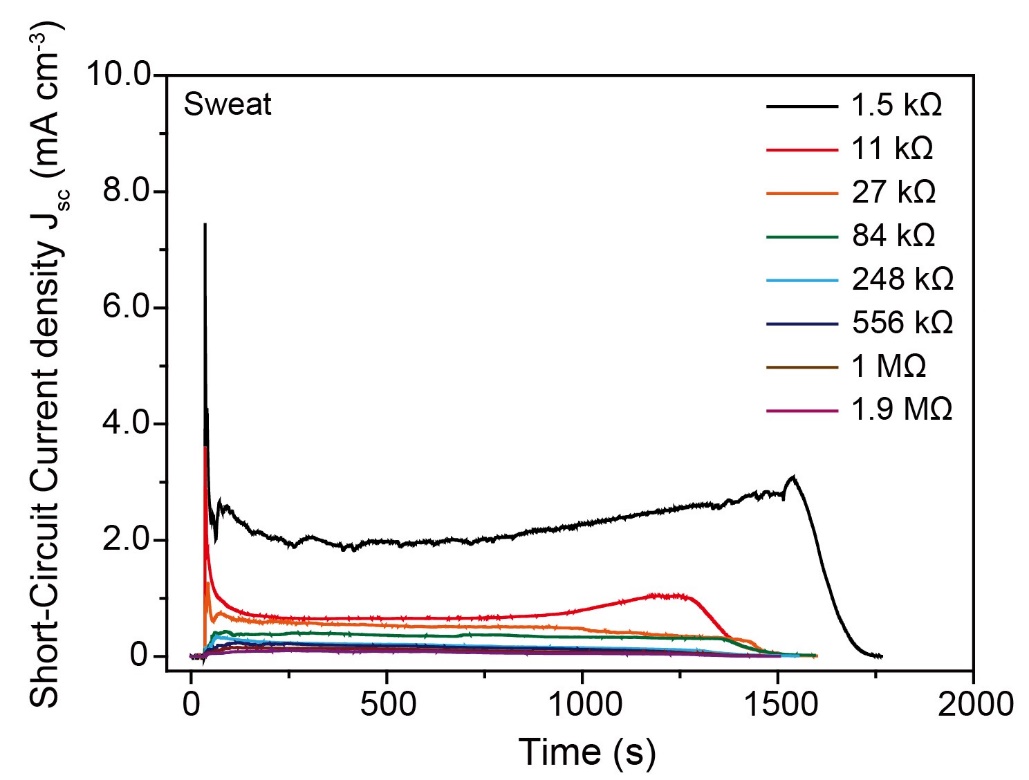


**Figure S19.** Measured J_sc_ profiles obtained by dropping 5 μl of the sweat salt solution on PEDOT:PSS/DVS-coated yarn-type TEPG systems with various resistances.

**Figure S20.** (a) Digital image of the experimental system for measuring power generation performance under deformation. Measured (b) V_oc_ profiles and (c) J_sc_ profiles of PEDOT:PSS/DVS-coated yarn-type TEPG system (11 kΩ) by dropping sweat salt solution. (d) Comparison of maximum V_oc_ and J_sc_ values with and without bending.

**
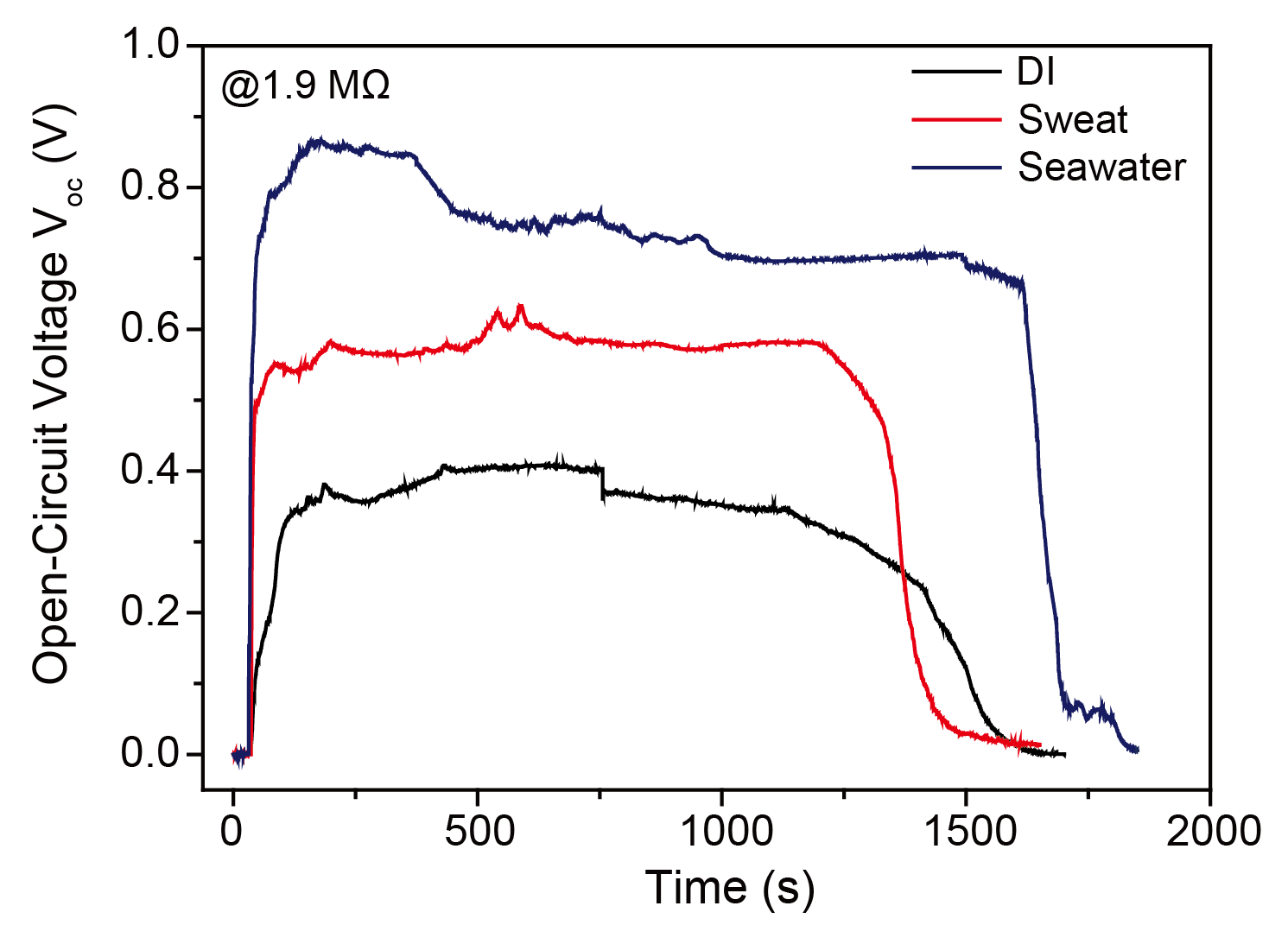
**

**Figure S21.** Measured V_oc_ profiles of PEDOT:PSS/DVS-coated yarn-type TEPG systems with various types of energy resources (1.9 MΩ).


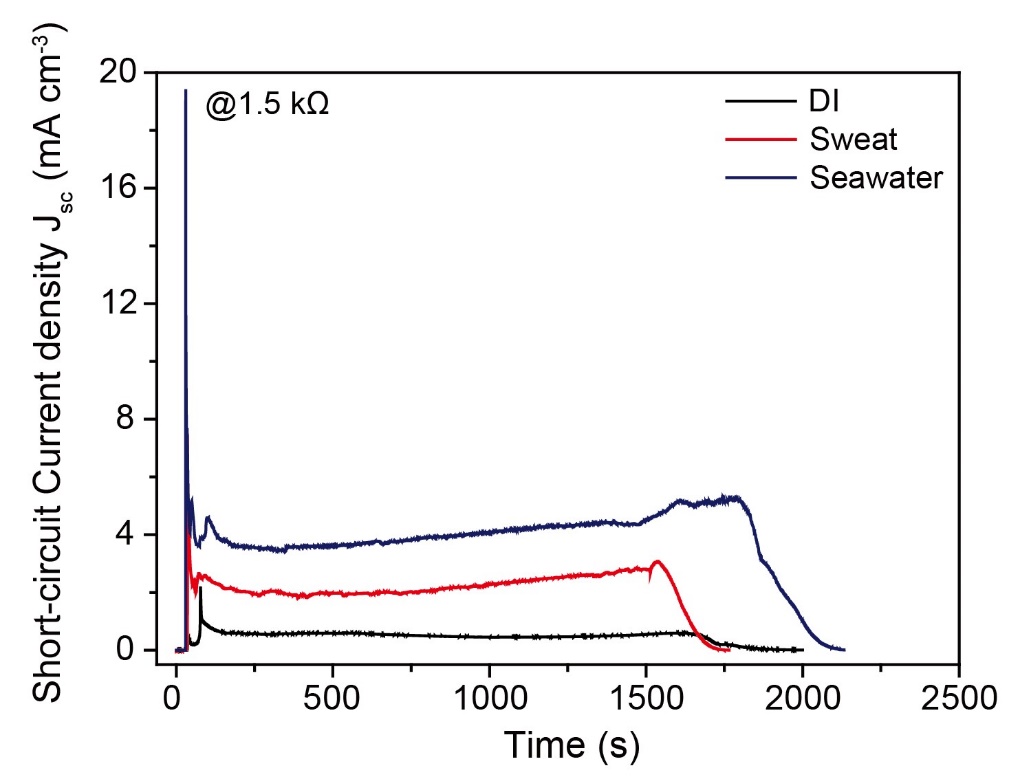


**Figure S22.** Measured J_sc_ profiles of PEDOT:PSS/DVS-coated yarn-type TEPG systems with various types of energy resources (1.5 kΩ).

**Figure S23.** Measured (a) V_oc_ and (b) J_sc_ profiles of PEDOT:PSS/DVS-coated yarn-type TEPG systems with various types of energy resources (11 kΩ).

**
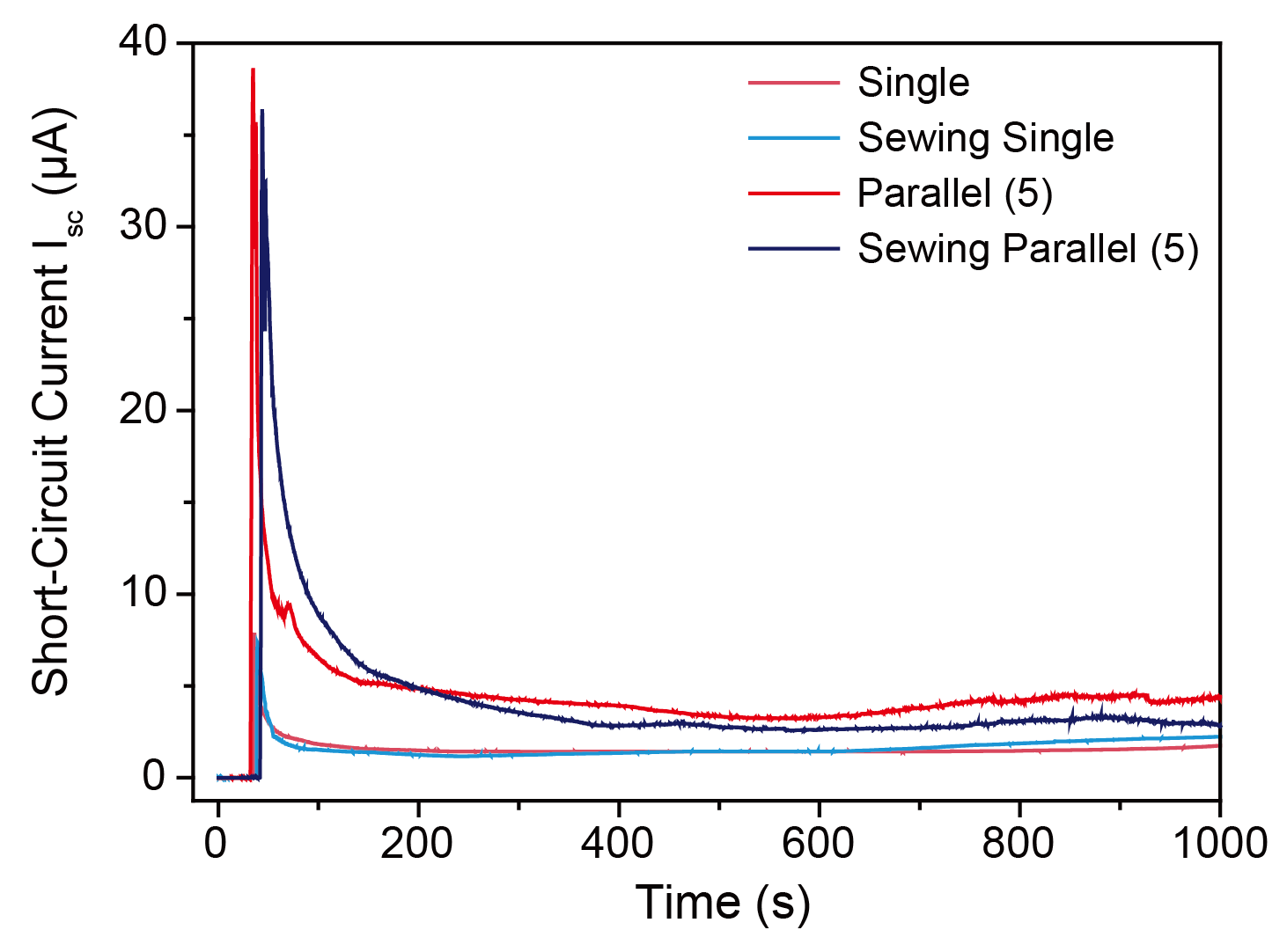
**

**Figure S24.** Measured I_sc_ profiles obtained by dropping 5 μl of the sweat salt solution on multiple PEDOT:PSS/DVS-coated yarn-type TEPG systems (11 kΩ) before and after sewing on waterproof textile in parallel.

**Table S1.** Comparison of power generation performance of various TEPG systems with different conductive absorbents.^[1-13]^

| No. | Material | Energy resource | V_oc_ | J_sc_ | P_max_ | Ref. |
| --- | --- | --- | --- | --- | --- | --- |
| 1 | Ketjen black | DI water | 0.53 V | 12.07 μA cm^−3^ | 0.79 μW cm^−3^ | [1] |
| 2 | Ketjen black | 35 g/L of NaCl salt solution | 0.174 V | 87.41 μA cm^−3^ | 3.8 μW cm^−3^ | [2] |
| 3 | Ketjen black | Saturated CaCl_2_ salt solution | 0.74 V | 69.44 μA cm^−3^ | 6.23 μW cm^−3^ | [3] |
| 4 | Carbon ink | 5M NaCl salt solution | 0.35 V | 5.65 μA cm^−2^ | 0.49 μW cm^−2^ | [4] |
| 5 | Carbon ink | 1M LiCl | 0.56 V | 6 μA cm^−3^ | 0.84 μA cm^−3^ | [5] |
| 6 | Oxidized CNT  /Oxidized carbon black | 1M NaCl salt solution | 0.75 V | 180.19 μA cm^−3^ | 33.79 μW cm^−3^ | [6] |
| 7 | CNT | 0.051 M NaCl salt solution | 0.16 V | 2.494 mA cm^−3^ | 100 μW cm^−3^ | [7] |
| 8 | Graphene  /Ketjen black | DI water | 0.21 V | 0.47 μA cm^−2^ | 0.025 μW cm^−2^ | [8] |
| 9 | MoS_2_ | 3M NaCl salt solution | 0.52 V | 91.31 μA cm^−3^ | 11.88 μW cm^−3^ | [9] |
| 10 | MXene/GO^a)^/CNT  /CNF^b)^/PVA^c)^ | 0.5 M NaCl salt solution | 0.69 V | 300.6 μA cm^−3^ | 51.85 μW cm^−3^ | [10] |
| 11 | PPy^d)^/TiO_2_ | DI water | 0.65 V | 3.64 μA cm^−3^ | 0.59 μW cm^−3^ | [11] |
| 12 | PPy/GO | 3.5 wt% of NaCl salt solution | 0.85 V | 4.44 μA cm^−2^ | 0.94 μA cm^−2^ | [12] |
| 13 | PEDOT:PSS | 0.4 M NaCl salt solution | 0.74 V | 220 μA cm^−3^ | 40.7 μW cm^−3^ | [13] |
| 14 | PEDOT:PSS/  DVS^e)^ | DI water | 0.116 V | 1.39 μA cm^−2^ | 0.04 μW cm^−2^ | This work |
|  |  |  |  | 236 μA cm^−3^ | 6.84 μW cm^−3^ |  |
|  |  | 0.051 M NaCl salt solution | 0.124 V | 21.3 μA cm^−2^ | 0.66 μW cm^−2^ |  |
|  |  |  |  | 3610 μA cm^−3^ | 111.91 μW cm^−3^ |  |
|  |  | 0.4 M NaCl salt solution | 0.255V | 34.22 μA cm^−2^ | 2.18 μW cm^−2^ |  |
|  |  |  |  | 5800 μA cm^−3^ | 369.75 μW cm^−3^ |  |

^a)^ GO – graphene oxide; ^b)^ CNF – cellulose nanofibers; ^c)^ PVA – poly(vinyl alcohol); ^d)^ PPy – polypyrrole; ^e)^ DVS – divinyl sulfone

**Supplementary references**

[1] T. G. Yun, J. Bae, A. Rothschild, I.-D. Kim, *ACS nano* **2019**, 13, 12703.

[2] Y. Li, S. Jiao, Y. Dai, J. Wang, J. Li, N. Kang, M. Irfan, X. Liu, *Appl. Energy* **2023**, 341, 121110.

[3] J. Bae, T. G. Yun, B. L. Suh, J. Kim, I.-D. Kim, *Energy Environ. Sci.* **2020**, 13, 527.

[4] Y. Lv, F. Gong, H. Li, Q. Zhou, X. Wu, W. Wang, R. Xiao, *Appl. Energy* **2020**, 279, 115764.

[5] F. Gong, H. Li, Q. Zhou, M. Wang, W. Wang, Y. Lv, R. Xiao, D. V. Papavassiliou, *Nano Energy* **2020**, 74, 104922.

[6] G. Luo, J. Xie, J. Liu, Y. Luo, M. Li, Z. Li, P. Yang, L. Zhao, K. Wang, R. Maeda, *Small* **2024**, 20, 2306318.

[7] J. Chen, Y. Li, Y. Zhang, D. Ye, C. Lei, K. Wu, Q. Fu, *Adv. Funct. Mater.* **2022**, 32, 2203666.

[8] R.-Y. Zhang, M. Gao, W.-R. Liu, W.-H. Chiang, L.-H. Yeh, *Carbon* **2023**, 204, 1.

[9] P. Wen, J. Ren, S. Ling, *ACS Appl. Electron. Mater.* **2023**, 5, 2082.

[10] Z. Yu, J. Mao, Q. Li, Y. Hu, Z. Tan, F. Xue, Y. Zhang, H. Zhu, C. Wang, H. He, *Langmuir* **2024**, 40, 5183.

[11] J. Xie, Y. Wang, S. Chen, *Chem. Eng. J.* **2022**, 431, 133236.

[12] Z. Guo, L. Lin, J. Ma, Y. Wang, T. Mei, X. Wang, *J. Mater. Res. Technol.* **2023**, 27, 2779.

[13] T. G. Yun, J. Bae, H. G. Nam, D. Kim, K. R. Yoon, S. M. Han, I.-D. Kim, *Nano Energy* **2022**, 94, 106946.
